# Identifying patterns in the multitrophic community and food-web structure of a low-turbidity temperate estuarine bay

**DOI:** 10.1038/s41598-020-73628-6

**Published:** 2020-10-06

**Authors:** Hee Yoon Kang, Changseong Kim, Dongyoung Kim, Young-Jae Lee, Hyun Je Park, Goutam K. Kundu, Young Kyun Kim, Riaz Bibi, Jaebin Jang, Kwang-Hun Lee, Hyun-Woo Kim, Sung-Gyu Yun, Heeyong Kim, Chang-Keun Kang

**Affiliations:** 1 School of Earth Sciences and Environmental Engineering, Gwangju Institute of Science and Technology, Gwangju, 61005 Republic of Korea; 2 Department of Marine Bioscience, Gangneung-Wonju National University, Gangneung, 25457 Republic of Korea; 3 Department of Marine Biology, Pukyong National University, Busan, 48513 Republic of Korea; 4 Department of Science Education, Daegu University, Gyeongsan, 38453 Republic of Korea; 5 South Sea Fisheries Research Institute, National Institute of Fisheries Science, Yeosu, 59780 Republic of Korea

**Keywords:** Environmental sciences, Ocean sciences

## Abstract

Food web dynamics outline the ecosystem processes that regulate community structure. Challenges in the approaches used to capture topological descriptions of food webs arise due to the difficulties in collecting extensive empirical data with temporal and spatial variations in community structure and predator–prey interactions. Here, we use a Kohonen self-organizing map algorithm (as a measure of community pattern) and stable isotope-mixing models (as a measure of trophic interaction) to identify food web patterns across a low-turbidity water channel of a temperate estuarine-coastal continuum. We find a spatial difference in the patterns of community compositions between the estuarine and deep-bay channels and a seasonal difference in the plankton pattern but less in the macrobenthos and nekton communities. Dietary mixing models of co-occurring dominant taxa reveal site-specific but unchanging food web topologies and the prominent role of phytoplankton in the trophic base of pelagic and prevalent-detrital benthic pathways. Our approach provides realistic frameworks for linking key nodes from producers to predators in trophic networks.

## Introduction

Estuaries form an intermediate transition zone between rivers and seas^1^. These coastal ecotones are characterized by highly dynamic physical, biogeochemical, and biological processes, and the functioning of these transitional ecosystems involves the complex interactions of those processes^2,3^. Fluctuations in the processes create distinct community patterns along the salinity gradient and thus the scale and magnitude of community patterns are subject to seasonal changes in freshwater discharge^4–6^. This wide spectrum of variability with space and time constructs the structural and dynamic properties of estuarine food webs^7,8^. Consequently, the understanding of food web dynamics in estuaries is fundamental for predicting the responses of communities (and populations) under both natural and anthropogenic forcing.

The identification of patterns in structure (e.g., links between species) and processes (e.g., energy or matter flux, interaction strengths) within food webs is crucial for understanding function and further constructing quantitative food webs for more detailed predictions of ecosystem-level responses^9,10^. Indeed, diverse network models have been applied to quantitatively analyse matter flows through trophic pathways within and among estuarine ecosystems, providing useful tools for assessing response patterns to environmental stresses^11–13^. To construct better food web models, it may be necessary to have empirical information on the underlying architecture (the map of predator–prey interactions) and interaction strength^8,14^. An estimation of the abundance and biomass of organisms often serves as a key component in computing the production of trophic groups^8^ and identifying patterns of co-occurrence of different trophic groups of organisms that create different types of interactions (i.e., competition, mutualism, and consumption) in a real community^15^. In addition, quantitative data on the diets of species are essential in terms of deciding trophic nodes in the food web and for partitioning the total consumption of each node into the relative contributions by other nodes, characterizing the sets of feeding links within a food web^16,17^.

Seasonal and longitudinal patterns in community structure in an estuarine-coastal continuum allow for the classification of benthic and pelagic community compositions along steep environmental gradients and the establishment of a solid basis of temporal and spatial scales for the further modelling of food webs. Spatial and temporal variation in typology within estuaries gives rise to concomitant changes in predator–prey interactions, making the generalization of estuarine food webs inappropriate^16,18,19^. Furthermore, while benthic suspension feeders rely on diets from the overlying water column, demersal fish often feed more on benthic prey than on pelagic prey^20,21^. Diets of predators also display ontogenetic as well as seasonal changes reflecting size selectivity and prey availability^19,22^. As a result, analyses of community composition should consider almost all taxonomic and/or functional groups colonizing both water and sediment at the appropriate spatial and temporal scales because of their potential interactions.

A variety of ordination techniques have been used to simply compile community patterns from highly complex community data. Considering the prevalent non-linearity of biological processes in estuaries^23,24^, we conveyed the abundances of phyto- and zooplankton, benthos, and nekton to the Kohonen self-organized map (SOM) algorithm^25,26^ to characterize community patterns along a salinity gradient that creates unique physical, chemical, and biological features. The SOM is an unsupervised neural network that has been widely applied to patterning communities^15,27,28^. The SOM finally performs clustering analysis of the input data through competitive learning and visualizes species assemblages on a bi-dimensional plane.

Once typological characteristics with time and space were determined, we used carbon and nitrogen stable isotope ratios of flora and fauna to infer the trophic relationships and the flow pattern of energy through the food webs^29^. This approach has been employed as an alternative tool for obtaining direct empirical information on consumer diets (e.g., gut contents). δ^13^C and δ^15^N have been commonly used to provide time- and space-integrated insights into trophic relationships between organisms, allowing for a comprehensive review of a priori assumptions of the trophic roles of individual organisms^29^. δ^13^C values can be used to trace original sources of dietary carbon because primary producers have distinct values from each other, and these values are conservative during trophic transfers, with little or no trophic enrichment (≤ 1‰)^30^. In contrast, the δ^15^N values of consumers manifest significant trophic-step fractionation in ^15^N (2–4‰, average 3.4‰ heavier than those of their prey) and are thus used as an estimate of the trophic position of consumers and the food chain length^31^. Estuarine ecosystems function in association with the large detrital pool derived from various types of vegetation^32^. Therefore, this approach is often more effective than other traditional empirical techniques in tracing the trophic pathways of carbon/energy and the trophic connectivity between habitats within estuaries^33^.

**Figure 1:**
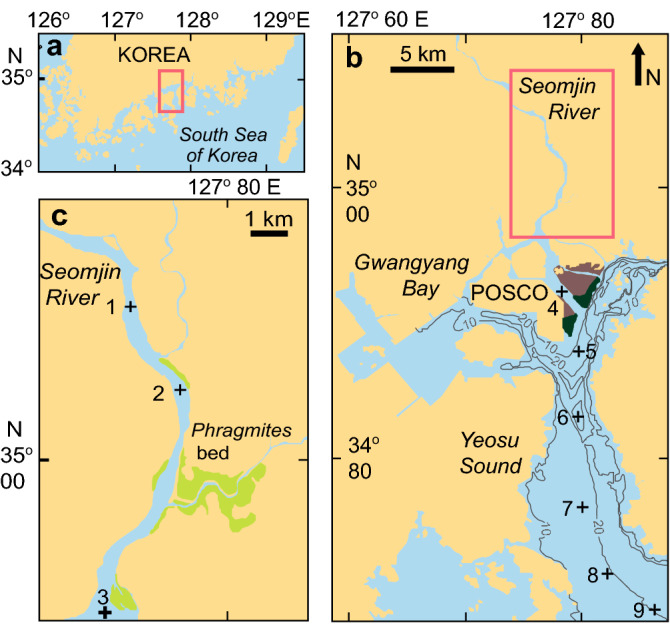
Map of study location and river-estuary-coastal sea continuum. (**a**) Map showing the location of Gwangyang Bay, Korea. (**b**) Sampling stations in the deep bay. The brown area represents the intertidal bed, and the dark green area represents the *Zostera* bed. (**c**) Sampling stations in the estuarine channel. The yellow-green area represents the *Phragmites* bed. The bay has an area of ca. 145 km^2^ and experiences a semidiurnal tidal cycle, with tidal ranges of 3.40 and 1.10 m during spring and neap tides, respectively. The Seomjin River discharges an annual mean of ca. 120 m^3^ s^−1^ (range: 30–95 m^3^ s^−1^ in winter base flows to 300–400 m^3^ s^−1^ in summer monsoon flows) of freshwater into the bay^34^. The northern estuarine channel is characterized by shallow depths (depth range: 2.4–8.0 m), short residence times (flushing time: 7.0 d)^35^, and low-turbidity water (extremely low concentrations, less than 20 mg l^−1^, of suspended particulate matter) following a lack of input of river-borne particles^36,37^. Three stations for the estuarine and six stations for the deep-bay channel were chosen for the field survey along a longitudinal water-channel trajectory of the estuarine-coastal marine continuum. See the Supplementary information for additional details.

Here, we combined the SOM algorithm and stable isotope techniques to explore the food web structure in the water channel along the estuarine-coastal marine continuum of a temperate coastal embayment, Gwangyang Bay, Korea, which is subject to low-turbidity riverine discharge and a short water residence time (Fig. 1). We synthesized multitrophic community patterns encompassing plankton, benthos, and nekton and determined subsets of co-occurring dominant taxa in different types of community associations with space and season. We then evaluated the relative contributions of dominant flora and river-borne organic matter in the landscape of the embayment to the dominant primary consumers (suspension and deposit feeders) that allowed for the subsequent calculations of the trophic links with benthic vs. pelagic pathways^29^. Using these mixing models, we integrated trophic interactions between main taxa that constitute key nodes of food-web networks. We found that a combination of both approaches enabled us to identify realistic food-web patterns at seasonal as well as spatial scales across the coastal ecotone.

## Results

### Patterning communities

**Figure 2:**
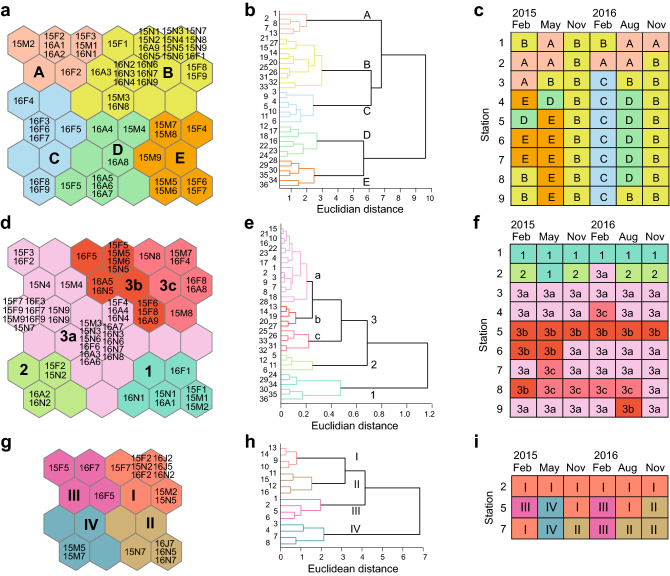
Patterning communities on the Kohonen self-organizing map (SOM). (**a**)**,** (**d**)**,** (**g**) Ordination of samples for plankton, benthos, and nekton, respectively, on the SOM. Acronyms of individual samples are denoted by a set of the sampling year (2015^15^ or 2016^16^), month (February [F], May [M], August [A], November [N]), the name of station (1–9). (**b**)**,** (**e**)**,** (**h**) Clustering of the trained SOM units. Numbers go from top to bottom and from left to right on the map. Clusters are indicated by different colours and corresponding numbers. (**c**)**,** (**f**)**,** (**i**) Distribution of clusters in time and space. A total of 225 taxa (species or genus) of phytoplankton, 66 of zooplankton, 77 of benthic invertebrates, and 105 of nekton (including fish, decapods and cephalopods) were identified. Among these, breakpoints of rank-frequency curves on each sampling occasion were first set to reduce the bias from rare species and left common taxa accounting for at least 5% of total abundances. Considering differences in mobility and sampling units between planktonic, benthic, and nektonic assemblages, community analyses were conducted separately. As a result, 26 phytoplankton, 28 zooplankton and 25 macrobenthic invertebrates, 17 nektonic invertebrates, and 15 fish taxa were selected to analyse community patterns.

Based on 54 individual datasets (9 sites × 6 times) of plankton assemblages (Supplementary Table 1), we trained the SOM and condensed them on the map (6 × 6 rectangular grid, Fig. 2a). The map configuration supported a clear segregation of seasonal samples from distributions in different areas on the map as well as a spatial separation of the estuarine stations in the upper left part of the map from the deep-bay stations. A hierarchical cluster analysis classified the SOM units into five groups (A, B, C, D, and E; Fig. 2b) that showed significant differences in terms of species composition (multi-response permutation procedure [MRPP], *A* = 0.10 to 0.42, *P* < 0.03 for all cases). The association between samples (individual datasets collected at a given site and time) and clusters (to which samples belong) depicted the seasonal and spatial patterns of clusters (Fig. 2c). In the estuarine channel (stations 1–2), the plankton assemblages displayed a seasonal succession alternating between clusters A and B, driving the differentiation from the deep-bay clusters (C, D, and E). The plankton assemblages in the deep bay (stations 3–9) showed a clear seasonal succession. In February and May 2015, most samples belonged to cluster E. The dominant cluster was replaced in turn by clusters B (November), C (February 2016), D (August), and B (November). Interestingly, plankton community at station 3, which is located at the mouth of the estuarine channel, alternated between estuarine and deep-bay clusters, forming the estuarine–coastal transition or mixing zone.

**Table 1:** δ^13^C and δ^15^N values, dependence on benthic affinity prey (*f*), and trophic position (TP) of dominant predatory consumers.

Taxon	Estuarine channel				Deep bay			
	δ^13^C (‰)	δ^15^N (‰)	*f*	TP	δ^13^C (‰)	δ^15^N (‰)	*f*	TP
**Macrobenthos**								
*Glycera chirori*					− 15.9 ± 0.7	13.6 ± 0.7 (14)	0.82	3.0
*Sigambra tentaculata*					− 16.1	13.2 (1)	0.75	2.9
*Scoletoma longifolia*					− 16.1 ± 0.8	12.9 ± 0.5 (5)	0.75	2.8
**Crustaceans**								
*Charybdis japonica*	− 17.2 ± 0.2	13.9 ± 0.7 (4)	0.58	3.2				
*Crangon hakodatei*	− 17.4 ± 0.7	12.9 ± 0.6 (3)	0.38	2.9				
*Oratosquilla oratoria*					− 15.5 ± 0.7	13.2 ± 0.4 (6)	0.96	2.8
*Charybdis bimaculata*					− 15.7 ± 0.4	12.0 ± 0.2 (5)	0.89	2.5
*Parapenaeopsis tenella*					− 16.0 ± 0.5	12.0 ± 0.4 (7)	0.79	2.5
*Metapenaeus joyneri*					− 16.0 ± 0.3	11.9 ± 0.4 (6)	0.79	2.5
*Trachysalambria curvirostris*	− 17.3	13.2 (1)	0.51	3.0	− 14.4 ± 0.6	12.6 ± 0.8 (5)	1.00	2.6
*Portunus trituberculatus*					− 15.0 ± 0.4	12.8 ± 0.4 (4)	1.00	2.7
*Palaemon gravieri*					− 14.4 ± 0.1	13.5 ± 0.4 (2)	1.00	2.9
*Alpheus japonicus*					− 14.2 ± 0.4	12.9 ± 0.4 (3)	1.00	2.7
*Alpheus digitalis*					− 14.1 ± 0.5	12.6 ± 0.7 (4)	1.00	2.6
**Cephalopods**								
*Loligo japonica*					− 15.8 ± 0.3	14.3 ± 0.7 (3)	0.86	3.2
*Octopus variabilis*					− 15.5 ± 0.4	13.6 ± 0.6 (4)	0.96	3.0
*Euprymna morsei*					− 14.7 ± 0.8	13.0 ± 0.4 (4)	1.00	2.8
**Fish**								
*Pleuronectes yokohamae*	− 16.0 ± 0.1	15.4 ± 0.3 (3)	0.79	3.5				
*Leiognathus nuchalis*	− 18.5 ± 0.5	14.7 ± 0.4 (3)	0.29	3.2	− 16.2 ± 0.6	15.0 ± 0.9 (6)	0.71	3.5
*Konosirus punctatus*	− 16.1	13.3 (1)	0.78	2.9				
*Cynoglossus joyneri*					− 15.0 ± 0.5	13.6 ± 0.5 (8)	1.00	2.9
*Amblychaeturichthys hexanema*					− 15.1 ± 0.4	14.2 ± 0.4 (6)	1.00	3.1
*Pennahia argentata*					− 15.2 ± 0.6	14.9 ± 1.0 (6)	1.00	3.3
*Thryssa kammalensis*					− 17.7 ± 2.6	14.6 ± 1.1 (6)	0.18	3.5
*Okamejei kenojei*					− 14.4 ± 0.5	13.4 ± 0.9 (5)	1.00	2.9
*Ctenotrypauchen microcephalus*					− 15.7 ± 0.5	14.1 ± 0.2 (4)	0.89	3.1
*Johnius grypotus*					− 14.7 ± 0.1	14.4 ± 0.8 (4)	1.00	3.2

Differences in the δ^13^C values of predatory macrobenthic and nektonic taxa between the estuarine channel and the deep bay were significant (Student’s *t*-test, *t*_27_ =  − 4.436, *P* < 0.001) but the same pattern was not observed for the δ^15^N values (*t*_27_ = 1.182, *P* = 0.248). The δ^13^C values of nektonic taxa collected from the estuarine channel were located outside the range of the end-member values (i.e., benthic and pelagic baselines). Therefore, considering their active movement characteristics, the dependence (*f*) of nektonic migrants in the estuarine channel on deep-bay benthic affinity prey was calculated using the IsoSource mixing model, using the isotope values of both benthic and pelagic baselines in the estuarine channel and the deep bay as end-members, and the model provided the median values (see Supplementary Table 8). When *f* 1, the *f* value was assigned to 1. The TP was estimated in the same manner as was done for the deep-bay predatory consumers, based on the δ^15^N value calculated from the contributions of four (benthic and pelagic) baseline end-members.

After training the SOM, the map (6 × 6 neurons) allowed us to visualize the information in the 54 datasets of macrobenthos assemblages (Fig. 2d). The spatial distribution of the samples on the map revealed a longitudinal pattern from the estuarine-channel stations in the lower part of the map to the deep-bay stations in the upper part of the map, further clustering the SOM units into three major groups (clusters 1, 2, and 3; MRPP, *A* = 0.09 to 0.24, *P* < 0.01 for all cases, Fig. 2e). Cluster 3 contained the samples from the deep bay and was subdivided into three subclusters (3a, 3b, and 3c; MRPP, *A* = 0.09 to 0.24, *P* = 0.001 for all cases). The distributions of clusters reflected a clear longitudinal pattern with no perceptible seasonal changes in the macrobenthic community (Fig. 2f). The estuarine-channel stations were divided into clusters 1 and 2. Of the deep-bay stations (3–9) belonging to cluster 3, subclusters 3b and 3c corresponded mostly to stations 5 and 8, respectively.

When the SOM was trained on 16 datasets (3 sites × 6 times) of nekton assemblages, their community patterns were visualized on a two-dimensional map (4 × 4 neurons, Fig. 2g). A visual inspection of the SOM map primarily revealed a clear spatial pattern in the nekton community by an array of estuarine channel stations in the upper right corner and deep bay stations in the other regions. The cluster analysis classified those two groups (MRPP, *A* = 0.17 to 0.44, *P* < 0.02 for clusters I vs. II, III or IV, Fig. 2h) and further divided the deep-bay samples into the lower right (cluster II), the lower left (III), and the upper left (IV) corners. The sample-cluster association then clarified the spatial pattern of the nekton community (Fig. 2i). Cluster I included samples collected at the estuarine station throughout the year, whereas clusters II, III and IV corresponded to the assemblages sampled at the deep-bay stations in August-November, February, and May, respectively. Seasonal discrimination in community composition was non-significant between clusters II vs. IV (MRPP, *A* = 0.38, *P* = 0.067) and clusters III vs. IV (*A* = 0.30, *P* = 1.000); the comparison showed a difference between the groups II and III (*A* = 0.22, *P* = 0.022), of which the result was less clear than the spatial pattern.

The visualization of the spatial distribution patterns of planktonic, benthic, and nektonic taxa on the SOM units manifested the associations of species with clusters (Supplementary Fig. 1). As measured by the IndVal index^38,39^, 16 phytoplankton, 20 zooplankton, 15 macrobenthos, 13 nektonic invertebrates, and 8 fish of common taxa considered in the indicator species analysis were found to be significantly (*P* < 0.05) associated with one or more clusters of taxonomic groups to which the species belonged, enabling us to summarize the assemblages characterizing each cluster (Supplementary Table 2). The relative abundance (***A***) of a species in a site group over all site groups and the relative frequency of occurrence (***B***) of that species inside the target cluster group recorded higher values than 0.52 (*Diatoma* sp.) and 0.50 (*Tortanus dextrilobatus*), respectively, in the present investigation. The square root of the IndVal index ranged from 0.71 for zooplankton (*Evadne nordmanni*) to 1.00 for benthic bivalves (*Corbicula japonica*) and fish (*Thryssa kammalensis*). Only a few indicator taxa were associated with the estuarine-channel clusters (A and 1–2) and were differentiated from the taxa of the deep-bay counterparts in the corresponding taxonomic groups. In contrast, the deep-bay clusters shared many indicator species and had a few unique characteristic taxa.

**Figure 3:**
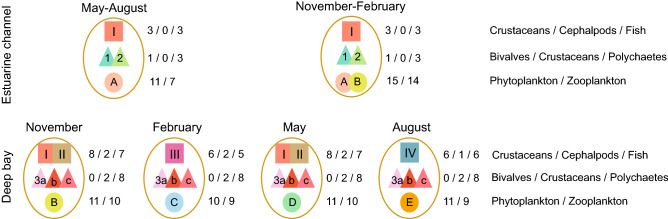
The associations of clusters and the number of dominant taxa constituting multitrophic communities. Based on the co-occurrence patterns at seasonal and spatial scales, we integrated sympatric clusters constituting plankton, benthos, and nekton communities during a given time period. The spatial characterization of the clusters was achieved between the estuarine channel and the deep bay but less clearly within the deep bay, except for the plankton assemblage in February 2015. The resultant associations of clusters generated multitrophic communities with arrays of two or four contrasting types for the estuarine channel and the deep bay, respectively, classifying the seasonal typology of biocenoses in respective localities. The number of dominant taxa in different taxonomic groups is indicated for cluster associations. The dominant taxa, expressed in numbers in the figure, are listed in Supplementary Table 3.

The association of dominant taxa^40^ in accordance with the configurations of cluster arrays illustrates the co-occurrence patterns of major food-web components within the multitrophic communities (Fig. 3, Supplementary Table 3). Clusters constituting the plankton community had an approximately equal number of dominant taxa (10–11 and 7–10 for phyto- and zooplankton, respectively). Although there were a few common dominant taxa in all clusters, there was a clear distinction in the dominant plankton taxa between the estuarine channel and the deep bay clusters. In the deep-bay clusters (B–E), some dominant taxa showed a varying frequency of occurrence with season. Clusters 1 and 2 of the macrobenthos community, which appeared in the estuarine channel, had only a restricted number (3 taxa for both) of dominant taxa compared to those (5–9 taxa) of deep-bay cluster 3. Three deep-bay subclusters shared the majority of dominant benthos taxa that created the clear separation from those in the estuarine clusters. Cluster I of the nekton community, present in the estuarine channel, recorded a lack of indicator taxa as well as a restricted number (6 taxa) of dominant taxa compared to those (13 taxa for each) in the deep-bay clusters II–IV. The median abundances of nektonic taxa in cluster I were much lower than those in the deep bay. The deep-bay clusters II–IV shared the majority of dominant taxa, some of which had an occurrence frequency that varied with season.

### Environmental characterization

**Figure 4:**
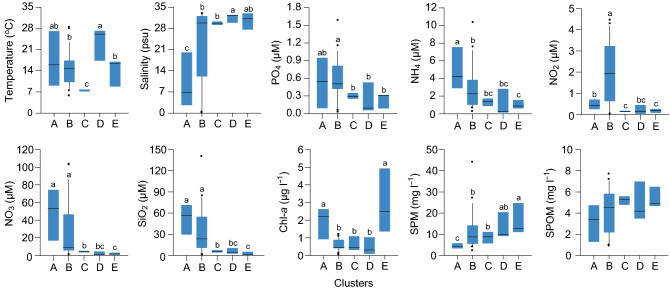
Environmental attributes of the water column in five clusters of plankton assemblages. Box-and-whisker plots of temperature, salinity, phosphate (PO_4_), ammonium (NH_4_), nitrate (NO_2_), nitrate (NO_3_), silicate (SiO_2_), chlorophyll *a* (Chl-*a*), suspended particulate matter (SPM), and particulate organic matter (SPOM) in the water column of five clusters (groups of stations). The median value of each cluster is displayed with the horizontal bar inside the box. Since cluster groupings of the plankton community represented seasonal as well as spatial patterns, we tested significant differences in environmental variables of the water column among those clusters using a Kruskal–Wallis test followed by a Mann–Whitney pairwise comparison test. The same superscript indicates a non-significant difference between medians (*P* > 0.05).

Comparisons of the environmental attributes of the water column were made among the values of the measured variables for the five clusters of plankton communities that represented both seasonal and spatial patterns (Fig. 4). A Kruskal–Wallis test identified significant differences (*H*-test, *P* < 0.001) in most variables. The following Mann–Whitney pairwise comparison test (at *P* = 0.05) revealed that the temperature peaked in the summer cluster D and was lowest in the winter cluster C; salinity was lowest in cluster A and consistently high in the other clusters (medians, > 29.5); dissolved inorganic nutrient concentrations were inversely related to salinity, being extremely high in clusters A and B and low in the other clusters; and chlorophyll *a* concentration displayed bimodal peaks in clusters A (estuarine channel) and E (spring). Suspended particulate matter concentrations were lowest in cluster A and highest in cluster E (with a median of 12.6 mg l^−1^). Most of the variables were characterized by broad variation ranges in clusters A (estuarine channel) and B (covering the whole bay area in fall) but by narrow ranges and comparatively low levels in the deep-bay clusters.

### Stable isotope measurements

**Figure 5:**
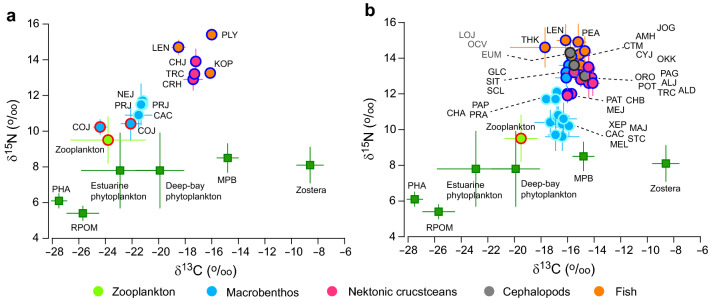
Biplots of δ^13^C and δ^15^N of primary sources of organic matter and dominant taxa. (**a**) The estuarine channel. (**b**) The deep bay. Strokes indicate the corresponding feeding mode: sky blue = deposit feeder, red = suspension feeder, dark blue = predator. List of dominant primary consumers: Bivalve *Corbicula japonica* (COJ); Crustaceans *Melita* sp. (MEL), *Xenophthalmus pinnotheroides* (XEP); Polychaetes *Neanthes japonica* (NEP), *Prionospio japonicas* (PRJ), *Capitella capitata* (CAC), *Magelona japonica* (MAJ), *Sternaspis scutata* (STS), *Paraprionospio pinnata* (PAP), *Chaetozone* sp. (CHA), and *Praxillella affinis* (PRA). Predatory taxa are listed in Table 1, and species codes are given by acronyms of first two letters of genus name and first letter of species name. The acronyms of organic matter sources are: *Phragmites australis* (PHA), locally produced (estuarine vs. deep-bay) phytoplankton, microphytobenthos (MPB), *Zostera marina* (Zostera), and riverine suspended particulate organic matter (RPOM).

Analysis of variance (ANOVA) revealed a great seasonal uniformity in the δ^13^C and δ^15^N values of wetland producers (*Phragmites australis*, microphytobenthos, and *Zostera marina*) and riverine suspended particulate organic matter (RPOM, *F*_3,40_ = 0.724 and 2.590, *P* = 0.544 and 0.066, respectively), which had ranges from − 27.5 ± 0.6‰ (annual mean ± 1 SD) to − 8.6 ± 1.0‰ and 5.4 ± 0.4‰ to 8.5 ± 0.8‰, respectively (*F*_3,40_ = 554.8 and 37.3, *P* < 0.001 for both; Fig. 5, Supplementary Table 4).

ANOVA and the following post hoc Tukey test (*P* < 0.05) indicated that the mean δ^13^C values of phytoplankton were clearly distinguished between clusters A–B (the estuarine channel, − 22.9 ± 2.6‰) and clusters B–E (the deep bay, − 19.9 ± 1.8‰) (*F*_5,44_ = 6.153, *P* < 0.001), while their δ^15^N values displayed undetectable seasonal and spatial variation (*F*_5,44_ = 0.403, *P* = 0.844), averaging 7.8 ± 2.1‰ (Fig. 5, Supplementary Table 5). ANOVA also showed that the δ^13^C values of zooplankton varied from a pooled mean − 23.8 ± 2.8‰ for clusters A–B (the estuarine channel) to one of − 19.5 ± 1.2‰ for clusters B–E (the deep bay) (*F*_5,38_ = 12.125, *P* < 0.001). The close proximity (a post hoc Tukey multiple comparison, *P* > 0.05) of the δ^13^C values between clusters in each location exhibited no seasonality. ANOVA revealed a non-significant difference in the δ^15^N values of zooplankton among clusters (*F*_5,44_ = 1.371, *P* = 0.257), with an overall mean of 9.5 ± 1.3‰. These area-pooled mean δ^13^C and δ^15^N values of zooplankton were used as isotopic baselines of the pelagic pathway in the subsequent isotope-mixing model for higher-level consumers.

The mean δ^13^C values of the dominant primary consumers (suspension feeders and deposit feeders) in each cluster of the macrobenthos community ranged from − 24.4 ± 0.5‰ to − 15.9 ± 0.5‰ (Supplementary Table 6). Their mean δ^15^N values varied little, ranging from 10.2 ± 0.4‰ to 11.8‰. Suspension feeders in the estuarine channel (Clusters 1–2) had slightly more ^13^C- and ^15^N-depeleted values (− 24.4 ± 0.5‰ to − 22.1 ± 0.7‰ and 10.2 ± 0.4‰ to 10.4 ± 0.9‰, respectively) than those (− 21.5 ± 1.0‰ to − 21.2 ± 0.4‰ and 10.9 ± 0.9‰ to 11.5 ± 1.2‰, respectively) of deposit feeders (Mann–Whitney U test, U = 6.5, *P* < 0.001 for δ^13^C; Student’s *t* test, *t*_18_ =  − 3.737, *P* = 0.002 for δ^15^N, Fig. 5a). Both δ^13^C and δ^15^N values of deposit feeders in the deep-bay clusters (3a–c) fell within very narrow ranges of − 16.7‰ to − 15.9 ± 0.5‰ and 9.6 ± 0.8‰ to 12.1‰, respectively (Fig. 5b). A clear spatial shift in the δ^13^C and δ^15^N values was found for deposit feeders between the estuarine channel (Clusters 1–2, mean − 21.3 ± 0.6‰ and 11.4 ± 0.6‰, respectively) and the deep bay (Clusters 3a–c, − 16.7 ± 0.8‰ and 10.5 ± 0.8‰) (Student’s *t* test, *t*_42_ = 16.637, *P* < 0.001 for δ^13^C; *t*_42_ =  − 2.990, *P* = 0.005 for δ^15^N). These area-pooled mean δ^13^C and δ^15^N values of deposit feeders were applied as isotopic baselines of the benthic pathway in the isotope-mixing model for higher-level consumers.

The mean δ^13^C values of dominant predatory taxa of macrobenthos and nekton clusters exhibited a significant spatial difference between the estuarine bay and the deep bay (Student’s *t*-test, *t*_27_ =  − 4.436, *P* < 0.001, Table 1). In the deep bay, their δ^13^C values were very consistent between taxa, with a narrow range from − 16.2 ± 0.6‰ to − 14.1 ± 0.5‰ (with only the exception of − 17.7 ± 2.6‰ observed for *T. kammalensis*), overlapping with the range of the deep-bay deposit feeders (Fig. 5b). The δ^13^C values of dominant nektonic taxa in the estuarine channel varied from − 18.5 ± 0.5‰ to − 16.0 ± 0.1‰, being, on average, 1.7‰ more negative than those of the deep-bay taxa but closer to the values of primary consumers of the deep bay than those of the estuarine channel (Fig. 5a).

### Isotope mixing model

**Figure 6:**
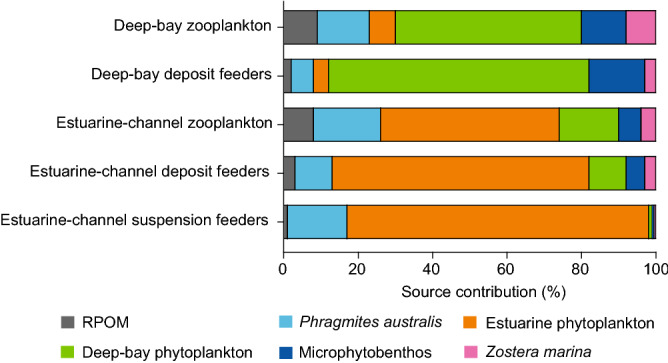
Feasible contributions (%) of primary producers to the nutrition of primary consumers. Primary producers include estuarine-channel suspension feeders, estuarine-channel deposit feeders, estuarine-channel zooplankton, deep-bay deposit feeders, and deep-bay zooplankton. The dominant primary producers considered include *Phragmites australis*, locally produced (estuarine vs. deep-bay) phytoplankton, microphytobenthos, *Zostera marina*, and riverine suspended particulate organic matter (RPOM). Feasible contributions represent median values. 1–99 percentile ranges for the distribution of feasible contributions of each source are given in Supplementary Table 7. Detailed procedures for estimation are given in the Methods.

The IsoSource mixing model calculation revealed that estuarine phytoplankton played a dominant role as the main nutritional contributor (median 81% [range: 74–86%] to suspension feeders, 68% [27–100%] to deposit feeders, and 46% [2–91%] to filter-feeding zooplankton) to the dominant primary consumers in the estuarine channel (Fig. 6, Supplementary Table 7). *Phragmites* is the next most influential contributor (16% [10–20%]) to food for the suspension feeders, but *Phragmites* and deep-bay phytoplankton share overlapping distributions of potential contributions (10% [0–30%] and 9% [0–54%], respectively, to deposit feeders; 18% [0–46%] and 14% [0–72%], respectively, to zooplankton) to the primary consumers. Riverine SPOM, microphytobenthos and *Zostera*, were minor nutritional contributors to estuarine channel consumers. In the deep bay, deep-bay phytoplankton generated the most important contributions of 69% (22–97%) and 47% (4–87%) to the nutrition of deposit feeders and zooplankton, respectively. Microphytobenthos made a considerable contribution of 14% (0–49%), but the others (riverine SPOM, *Phragmites*, estuarine phytoplankton, and *Zostera*) were only minor contributors (medians > 2–6%) to the nutrition of deposit feeders. The nutritional contributions of riverine SPOM, *Phragmites*, estuarine phytoplankton, and *Zostera* to the deep-bay zooplankton were equally substantial, with values of 8% (0–38%), 13% (0–44%), 6% (0–36%), 11% (0–54%), and 7% (0–34%), respectively.

Using the δ^13^C and δ^15^N values of the deep-bay zooplankton and deposit feeders as the isotopic baselines of benthic and pelagic trophic pathways, our two-source mixing model calculations revealed that higher-level (predatory) consumers exclusively prey (71–100%), with only the exception of 18% of *T. kammalensis*, on benthic affinity prey in the deep bay (Table 1). The δ^13^C values of the nektonic taxa collected from the estuarine channel were located outside the range of the end-member values (i.e., the benthic and pelagic baselines). Therefore, considering the motile characteristics of nekton influenced by tidal movement, the dependence (*f*) of the estuarine-channel nekton on the deep-bay benthic affinity prey was calculated using the IsoSource mixing model based on both benthic and pelagic baseline values in the estuarine channel and the deep bay as end-members. The deep-bay benthic affinity prey was estimated to play an important role (median values of 51–79%) as dietary contributors to the estuarine-channel motile consumers, with the exception of *Crangon hakodatei* (38%) and *Leiognathus nuchalis* (29%), which had dependence values of 49% and 62% on the deep-bay pelagic and the estuarine-channel benthic affinity prey, respectively.

Dominant predatory consumers displayed an average dietary trophic position estimate between 2.5 and 3.5 in the estuarine and deep-bay channels (Table 1). Trophic position values of macrobenthos and crustaceans fell within the range from 2.5 to 3.2, making them omnivorous and secondary consumers. The average trophic position estimates of fish ranged from 2.9 to 3.5. being on average ~ 0.4 higher than those of macrobenthos and crustaceans. The observed spatial variation in fish trophic position exhibited a high degree of overlap in their trophic position between estuarine and deep-bay channels.

## Discussion

While a large variety of theoretical stimulations and empirical tests have been adopted to construct community trophic networks, identifying key species and feeding links is crucial to the architecture of a food web diagram that further characterizes ecological dynamics^9,41,42^. We combined community patterns with stable isotope determination to characterize typologies in a community-wide food web structure in a temperate estuarine embayment. Our community pattern analyses, based on the SOM procedure, revealed distinct typological patterns between the estuarine channel and the deep bay as well as differing seasonal patterns among plankton, benthos, and nekton assemblages. Subsequent isotope mixing-model estimation of trophic linkages between co-occurring dominant taxa (as key nodes of networks) highlighted a spatial variation in food web architecture that was in accordance with community typologies.

The plankton community was first divided into estuarine-channel and deep-bay clusters. The estuarine-channel clusters were clearly associated not only with the occurrence of freshwater, brackish, and benthic indicator taxa (e.g., *Diatom*a sp., *Nitzchia* sp., *Cylindrotheca closterium*, *Sinocalanus tenellus*, and *T. dextrilobatus*)^43,44^ but also with low salinity and high inorganic nutrient and chlorophyll *a* concentrations. Along with the seasonal reduction of river discharge in November and February, the plankton community in the estuarine channel shifted towards deep-bay cluster B. The sample-cluster association further clarified the importance of seasonality in the transition of the deep-bay plankton community. The spatial uniformity of the plankton community in each season and the prevalence of marine taxa in indicator species provide clear indications of marine ecosystem attributes^45^. Consistently high salinity and low inorganic nutrient concentrations and turbidity in the deep bay reflect that river discharge is rapidly diluted with and diffused into inflowing marine water that is highly dynamic and quickly renewed owing to the short water residence time of 2.5–3.4 d driven by semidiurnal tides^46^. Previous studies also proposed seasonal succession in the phytoplankton community (i.e., relative abundances of centric and pennate diatoms and flagellates) in response to temperature fluctuation, inorganic nutrient loading status and N:P stoichiometry^47^ as well as zooplankton assemblages linked to temperature, salinity and *Noctiluca* vs. diatom densities^43,48^.

Our SOM training intuitively differentiated the macrobenthic community into clusters 1 and 2 (estuarine channel) and cluster 3 (deep bay), reflecting a salinity gradient^49^. The dominance of the brackish-water indicator species *C. japonica* and *Prionospio japonicus* in the estuarine-channel clusters supports the longitudinal transition of the macrobenthic community. Although patchy distributions of some indicator taxa in the central area (stations 5 and 8) of the deep bay created subclusters, many indicator and dominant taxa were widespread in deep-bay cluster 3 throughout the year, further highlighting a lack of seasonality in the succession of the macrobenthic community. Historically, the deep bay has experienced extensive anthropogenic activity, such as large-scale reclamation and dredging in the 1980s and 1990s, which led to changes in sediment granulometry (from sand to fine-grained clay and silt). Alterations in habitat features due to major geological events have modified the spatial distribution patterns and dominant taxa of macrobenthic assemblages^50^. Given that most macrobenthic taxa are sedentary with little mobility, seasonally persisting macrobenthic community patterns appear to reflect the stabilized sedimentary condition of the bay habitats^50^.

The nekton community patterns indicated the occurrence of four assemblage types that represent the spatial variation (estuarine channel vs. deep bay) and seasonal variation (within the deep bay). Cluster I, representative of the estuarine channel, was characterized by a very restricted number of dominant taxa without an assignment of an indicator species due to a random distribution (low frequency) and low abundances of species. Clusters II–IV mainly depicted the seasonal transition of the deep-bay nekton assemblages. However, our IndVal analysis highlights extensive overlap in indicator species between clusters (at least two clusters share 18 of 21 taxa), and further multi-response permutation procedure (MRPP) testing concludes less clear seasonal than habitat environment-based patterns as previously observed^51^. The observed seasonal succession of the deep-bay assemblages, although not great, seems to be attributed to the addition of a few seasonal (or temporary) migrants. As a result, the nekton assemblages were characterized by rare species in the estuarine channel and by the predominance of year-round resident species, which occupied over 80% of the total abundances^51,52^, with a few transient migrants in the deep bay.

The association of co-occurring clusters of plankton, benthos, and nekton assemblages illustrates multitrophic communities that can be formed by any type of interspecific interactions in a given site and time (Fig. 3). As discussed earlier, the distributions of indicator taxa vary systematically across the estuarine channel and deep bay, characterizing the spatial and seasonal organization of biocenoses in accordance with environmental conditions. Therefore, the association between the identified indicator taxa and clusters underpins the intrinsic linkage between the SOM clusters. While trophic networks have long been recognized as a key component in ecological systems, the estimation of food web functioning has been challenging because of the difficulties in measuring interactions between species that constitute the nodes of such networks^7,53^. In this context, while taxa with high IndVal values represent an indicator of particular environmental conditions, their role in trophic transfers would be equivocal because they are found at smaller abundances than dominant taxa^40^. Dominant taxa with high abundances and frequencies in the associations of clusters can be assigned to major trophic components that are most influential in community dynamics and constitute key nodes of multitrophic levels in complex ecological networks^15,42^.

Stable isotopes reveal trophism and trophic connections of the co-occurring dominant taxa in the multitrophic community. Consistent with the spatial characterization of the associations of co-occurring clusters, the occurrence of dominant taxa differed considerably between the estuarine channel and the deep bay. In the estuarine channel, the dominant plankton taxa varied between the summer wet and winter dry periods, but the macrobenthos and nekton taxa were seasonally identical with a very restricted number. Our isotope mixing-model calculations demonstrated that pelagic primary production forms the important base of the estuarine trophic network. This result is supported by the high chlorophyll *a* levels retained by pelagic and benthic diatoms throughout the year. High abundances of both herbivorous and predatory zooplankton complicate the planktonic food webs and links with other predators. Dominant zooplankton had consistent δ^13^C values with those of phytoplankton throughout the year. This result suggests that switching plankton assemblages guarantees the persistence of patterns of planktonic food chains (e.g., trophic base, resource utilization, and trophic niche)^43^.

**Figure 7:**
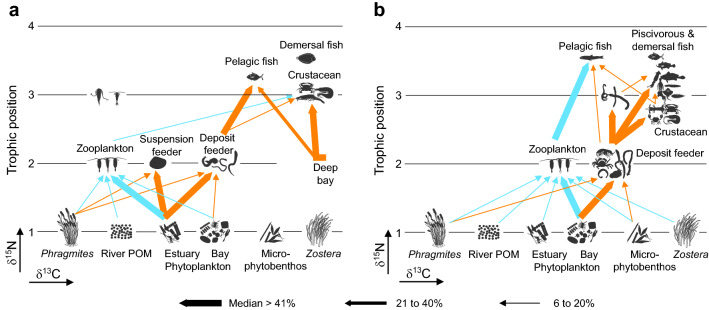
Representative food web of water channel community of Gwangyang Bay. (**a**) Estuarine channel. (**b**) Deep bay. Sizes of linkage arrows indicate the relative contribution of prey to consumer nutrition. Sky blue arrows indicate pelagic pathways and orange benthic pathways. Conceptual food-web models were generated for two longitudinally different communities, based on co-occurring dominant taxa in the associations of clusters constituting multitrophic communities presented in Fig. 3. Despite seasonal changes in community composition (largely plankton assemblages) and population size structure of certain species, most trophic connections and food-web pathways remain unchanged in two different localities. Dominant taxa in each trophic group are summarized in Supplementary Table 9.

The split alignment of δ^13^C values between primary consumers and dominant nekton in the estuarine channel indicated the lack of trophic connection between them. Indeed, our mixing-model calculations denoted that most of the nutrition of these motile consumers was derived from the deep-bay benthic prey (Table 1), most likely reflecting their rapid channel-bay movement due to the high tidal prism and short residence time^35,46^. An increase in the consumption of estuarine prey was found only for the fish *L. nuchalis*, which feeds mainly on benthic prey at the adult stage in the estuary^54^, and the shrimp *C. hakodatei*. The overall combination of the mixing-model calculations allowed us to draw a generalized food web structure that remains unchanged with season in the estuarine community (Fig. 7a). The low number and abundances of dominant nekton suggest that biomass transfer to higher-level consumers is modest, simplifying the higher-level feeding links in the channel community.

Our overall mixing-model calculations for the highly diversified deep-bay community are summarized by the seasonally consistent architecture of the food web in the deep-bay ecosystem (Fig. 7b). Chlorophyll *a* concentrations peaked in spring and remained at relatively low levels despite high photosynthetic activity^37^ during the rest of the year, supporting high grazing activity. Despite compositional changes in the dominant phytoplankton taxa, the consistent and close proximity in the δ^13^C values between bay phytoplankton and primary consumers throughout the year suggests a functional redundancy of phytoplankton assemblages, as was discussed for the estuarine channel. Indeed, our isotope mixing-model calculations emphasized the trophic importance of deep-bay phytoplankton to both benthic deposit feeders and zooplankton. The discrimination of the δ^13^C values between benthic and pelagic feeders may be explained by the feeding on ^13^C-enriched sources (e.g., sedimentation of phytoplankton blooms^55,56^ and zooplankton faecal pellets^57^ or some fractions of microbial and microalgal biomass^16,58^) by deposit feeders, taxon-specific differences in trophic fractionation of C isotopes^59,60^, and the degradation or microbial recycling status of sedimentary organic matter^61,62^.

The general pattern of the δ^13^C alignment and δ^15^N increase between the benthic and nektonic consumers reveals their strong trophic linkages, emphasizing the prevalence of benthic pathways. Furthermore, the relatively stabilized numbers and abundances of dominant benthic taxa reject seasonal dietary shifts of nektonic predators in response to changes in prey resource availability. The estimated trophic positions of motile predators indicate prevalent omnivory. Such a functional role of diverse benthic taxa as trophic mediators in the mid-trophic levels would attest to food-web stability in response to compositional changes in the lower trophic-level assemblages. The only exception of benthic feeding was observed for the planktivorous fish *T. kammalensis*^63^. An increased reliance of *L. nuchalis*, the most abundant fish, on benthic prey likely reflects an ontogenetic dietary shift from planktivorous feeding in the estuarine channel to feeding on polychaetes and crab larvae in the deep bay^54^.

To conclude, our results successfully differentiated two contrasting community structures between the estuarine channel and the deep bay. Our results also indicated that the autochthonous production of phytoplankton serves as a principal basal resource supporting the unvegetated, low-turbidity estuarine and deep-bay food webs, contrasting with diversified food-web bases found in marsh-covered or other highly turbid coastal systems^16,64,65^. Furthermore, food-web topologies persist across seasons in accordance with the great compositional consistency of benthic and nektonic communities in respective areas, posing a challenge to the formation of a general consensus of seasonality in the web structure^16^. It would be expected that ontogenetic changes in the food web components may change interaction strengths at the species level, adding complexity^9^. Various approaches for ecological network analysis have attempted to quantitatively assess the interactions among species (or guilds), their demographic effects and the dynamics of communities^7,66^. Our empirical analysis provides the basis of the food web structure to select nodes at the species and/or guild levels, construct networks, and quantify ecosystem functioning^58^; additionally, we were able to identify unchanging food-web architecture of merit for ecological modelling in our model system^67^. Finally, isotope measurements can partition primary source contribution to dominant detritivory in benthic pathways and provide potential to depict complete trophic transfer in end-to-end food webs (from microbes to large metazoans) by determining microbial compartments^16,58^.

## Methods

### Field samplings and data acquisition

Nine sampling stations were chosen along a main water-channel trajectory of the river-estuary-coastal sea continuum in the bay (Fig. 1, Supplementary information). Sampling and data collection were conducted seasonally (February, May, and November 2015, and February, August, and November 2016) at nine stations for two years. Sampling months were chosen as representatives of seasonal physical characteristics according to clear seasonal variations in precipitation, freshwater discharge, water temperature, and salinity typical of summer rainy and winter dry conditions, and spring and autumn transitional conditions in the temperate northeast Asian monsoon climate zone^37^. Data included physical and chemical parameters and abundances of phytoplankton, mesozooplankton, and benthic invertebrates. Nektonic (fish and invertebrate) samples were collected at only three locations (stations 2, 5, and 7) because of their migration characteristics and long field collection area (tens of metres). Additional collections of specimens for dominant taxa were conducted to analyse the stable isotope ratios. To acquire stable isotope data of potential sources of organic matter, suspended particulate organic matter (SPOM), the dominant marsh plant *Phragmites australis*, microphytobenthos, and the seagrass *Zostera marina* were also collected at respective locations.

On each sampling occasion, the water temperature and salinity were measured in situ using a YSI Model 85 probe (YSI Inc., Yellow Springs, OH, USA). The dissolved oxygen concentrations were high (over 6.5 mg l^−1^) in both the surface and the bottom layers throughout the year, with no hypoxia; thus, they were not reported in the present study. Water was collected using a 10 l van Dorn water sampler at the subsurface (1 m below the water surface) and at the bottom (variable with depth: 2.4–8.0 m in the northern estuarine channel and 10–30 m in the deep-bay channel) of each station. Water samples for suspended particulate matter (SPM), chlorophyll *a* (Chl *a*), and dissolved inorganic nutrient measurements were immediately prefiltered with a 180-μm Nitex mesh to eliminate large particles and zooplankton; samples were collected in acid-washed plastic bottles and stored in the dark on dry ice. For phytoplankton species abundances, 1 l of seawater collected was contained in glass dark bottles, fixed with Lugol's solution (3% of final concentration), and concentrated for later analysis. Additional details of the analytical procedures and adopted methods for the quantification of SPM (SPOM), Chl *a*, and dissolved inorganic nutrient concentrations and the identification of phytoplankton species after transportation of water samples to the laboratory are given in the Supplementary information.

Mesozooplankton samples were collected using a conical plankton net (mesh sizes 200 μm, mouth diameter of 45 cm) equipped with a Model 2030R Mechanical Flowmeter (General Oceanics Inc., Miami, FL). The net was obliquely towed several times from the bottom to the surface at a speed of 1 m s^−1^. After collection, the specimens were poured into 300 ml plastic bottles and immediately fixed with a 10% solution of borax-buffered formalin.

Macrobenthic invertebrates were collected by sieving sediment through a 1-mm mesh net after collection using a 0.12 m^2^ van Veen grab. Three to five replicates at each station were sampled to analyse the benthic community of the bay. Because of the dominance of fine sediments, sediments collected were broken up in water inside the bucket by adding seawater and stirring gently before sieving. The sieved material was placed in 2 l plastic containers and immediately preserved with a 10% solution of borax-buffered formalin. After a few days, the samples were removed from the fixing solution, rinsed and preserved in an ethanol solution (70% ethanol and 5% glycerin) in the laboratory.

Nekton (both fish and invertebrates) were collected using a beam trawl, the opening (mouth) of which was 8.5 m long and 1.1 m (maximum 3 m) high. The mesh size of the trawling net was 18 mm on the wings and belly of the net and 10 mm in the cod end. A much smaller mesh net than that used in commercial fishing gear (25–240 mm mesh net) was used to capture small specimens. Two replicates were conducted at each site for approximately 1 h per trawl set at a speed of 4.0 km h^−1^. All materials captured were placed in labelled plastic containers and fixed in 10% formalin as described above. The detailed laboratory procedures for the identification and numeration of animal samples are presented in the Supplementary information.

### Sample processing for isotopic measurement

Additional samplings for stable isotope analyses were conducted by adopting the same manner and time as used for the collection of environmental parameters and community analysis. For SPOM samples, approximately 20 l of water was collected at each station and a river discharge area using a van Dorn water sampler, prefiltered in situ with a 180-μm Nitex mesh, and filtered again onto the GF/F filter (ϕ25 mm). In the laboratory, the filter samples were acidified by fuming for 24 h in a desiccator saturated with HCl to remove the inorganic carbon^68^. Particulate organic carbon (POC) and nitrogen (PN) concentrations and δ^13^C and δ^15^N values were determined on filtered and dried (at 60 °C) particulate samples. To determine isotope ratios of micro-size phytoplankton such as diatoms, a conical plankton net (mesh size of 20 μm, mouth diameter of 45 cm) was vertically towed at each station. Some phytoplankton samples concentrated into individual test tubes were acidified by adding several drops of 0.12N HCl for δ^13^C measurements before drying at 60 °C, and those for δ^15^N were not acidified.

To detect a possible cross-boundary resource flux by the isotopic signatures of consumers, *Phragmites*, microphytobenthos, and *Zostera* samples were collected in the upper estuarine wetland, on the northern intertidal muddy sand flat, and on the fringe of the sand flat, respectively (Fig. 1). The *Phragmites* and *Zostera* leaves were collected by hand, and MPB was collected by scraping the visible mat of benthic diatoms from the sediment surface during low tide. Pure microphytobenthos was extracted by collecting benthic diatoms attached to silica powder^69^. These plant samples were washed with Milli-Q water and lyophilized at − 70 °C. To determine the stable isotope ratios of sedimentary organic matter, slices of the surface sediments (top 2 cm) were put into 20 ml glass tubes after collection using a van Veen grab and kept on dry ice in the dark. After thawing in the laboratory, sediment samples were treated with 10% HCl solution until the bubbling to remove carbonates stopped, oven dried for 72 h at 60 °C, and homogenized by pulverizing.

Zooplankton and macrobenthic invertebrates collected for isotope analysis were kept alive for at least 6 h in filtered seawater on board to allow them to evacuate their gut materials. All animal samples were submerged in cold (< 5 °C) water containers and transported to the laboratory. After sorting and identification, only muscle tissues of live and intact molluscs, crabs and shrimp of macroinvertebrates were collected to minimize contamination with other materials. Whole body tissues of polychaetes and only white muscle tissues of the dorsal region of fish were prepared after the removal of their viscera by dissection. They were not acid treated. Copepods, amphipods, and other small invertebrates, including crustaceans, gastropods, and bivalves, were pooled to provide enough material for isotope analysis. They were decalcified with 0.12N HCl solution until the bubbling stopped to remove the probable effects of carbonates. Another series of their tissues was not acid-treated for nitrogen isotopic analysis. The δ^13^C values of zooplankton and fish tissues displayed substantial shifts before and after lipid extraction in the present study, reflecting the species’ differences in their contents of isotopically lighter lipids^70^. We used the δ^13^C data for the defatted samples of zooplankton and fish tissues, and the detailed defatting procedures before analysis are given in the Supplementary information.

### Stable isotope analysis

All the pre-treated floral and faunal samples were kept frozen, lyophilized and then pulverized to a fine powder with a ball mill before analysis (Retsch MM200 Mixer Mill, Hyland Scientific, WA). Filters containing SPOM were wrapped with tin foil, and the powdered samples were packed into tin combustion cups (8 × 5 mm). For all sealed samples, the δ^13^C and δ^15^N values were measured using a continuous flow isotope ratio mass spectrometer (CF-IRMS; Isoprime, GV Instrument, Manchester, UK) connected to an elemental analyser (Eurovector 3000 Series, Milan, Italy). The carbon and nitrogen elemental concentrations were analysed simultaneously with isotopic composition. Isotopic values were expressed in the conventional delta as deviations from standards (i.e., Pee Dee Belemnite for carbon and atmospheric air for nitrogen) following the formula: $\delta{\text{X}} \left( \permil \right) = \left[ {\left( {R_{sample} /R_{standard} } \right) - 1} \right] \times 10^{3}$, where ${\text{X}}$ is the ^13^C or ^15^N and $R$ is the ^13^C/^12^C or ^15^N/^14^N ratio. Accuracy was established through repeated analyses of internal laboratory standards calibrated against International Atomic Energy Agency standards CH-6 (sucrose) and IAEA-N1 (ammonium sulphate). The instrument precision was 0.15‰ for δ^13^C and 0.2‰ for δ^15^N based on replicate measurements of these reference materials.

### Self-organizing map (SOM) modelling

A self-organizing map (SOM) with a Kohonen’s competitive and unsupervised artificial neural network algorithm, which approximates the probability density function of the input data^26^, was employed to characterize the distribution patterns of communities. The SOM performs a nonlinear projection of multivariate datasets onto a two-dimensional space. This algorithm constitutes two layers. In the present study, the input layer includes 54 nodes (one by taxa) connected to the sampling datasets of 9 sites × 6 times for plankton and benthos and 18 nodes of 3 sites × 6 times for nekton. The initialized virtual sites drawn from these input datasets are updated by an unsupervised learning procedure based on an iterative method. The subsequent learning phase determines the ‘best matching unit’ based on Euclidean distance, and the further ordering and tuning phases are repeated to adjust the neighbouring units. In the training phase, the weights of the whole neighbourhood are moved in the same direction. Finally, each input sample is linked to the corresponding hexagon (each unit) of the map. Neighbouring map units on the two-dimensional grid are similar and thus expected to be clustered together.

Consequently, the output layer is composed of 36 neurons for plankton and benthos, organized in a rectangular grid of 6 × 6, and 16 neurons for nekton, in a grid of 4 × 4. The number of output neurons was chosen by two approaches. The grid size was first chosen according to Vesanto’s heuristic rule^71^ of 5 $\sqrt N$, where N is the size of the dataset, and finally, the actual size of the SOM map was decided based on the minimum values of the quantization error (QE) and topographic error (TE) by running the entire procedure several times^72^. The QE and TE values were 0.110 and 0.037 for plankton, 0.108 and 0.074 for macrobenthos, and 0.148 and < 0.001 for nekton assemblages, respectively.

Training of the SOM and the clustering procedures were performed using MATLAB software (Version 6.1, MathWorks, Natick, MA). Prior to training, the species abundance data were scaled to a range of 0–1 to reduce the variation and skewness of abundance and normalized to the interval ZEW to impose the same weight on different taxa that appear in different ways (e.g., order, number, units). A unified-matrix displays the Euclidean distance between weight vectors of neighbouring units of the SOM and allows us to detect the cluster boundaries on the map (data not shown here). After training, Ward’s minimum variance method with the Euclidean distance measure was applied to a hierarchical cluster analysis of the SOM units^73^. The significance among the clusters was tested by a multi-response permutation procedure, which is a nonparametric method used to test differences between groups, using R 3.4.1 software (vegan library^74^). In addition, the trend of occurrence of each species in the clusters was displayed by the degree of colour on the SOM, making the importance of a given species to particular clusters identifiable.

### Indicator and dominant species

The visual map after the training of the SOM provides a trend of species occurrence instead of statistical indication and classifies map units into clusters that consist of similar assemblages. To identify species (or groups of species) that might play a very prominent role as an indicator of particular environmental conditions, we performed an indicator species analysis^38,39^. This approach allows us to determine an indicator value (IndVal) of species that may be obtained from the original data matrix (i.e., abundances) for the clusters discriminated by the SOM, considering both the dominance and the frequency of the species in the clusters. The IndVal index, which presents the association between species and groups of sites (clusters), was calculated by the product of the relative abundance (***A***) of a species (in a site group vs. all site groups) and the relative frequency of occurrence (***B***) of that species inside the target cluster group, and the significance of the relationship was tested by using a Monte Carlo permutation test^39^. The resulting association index returns the square root of the IndVal index (IndVal.g)^38^. The maximum indicator value (= 1) can be seen when all occurrences of a species are found in either individual groups of sites or a combination of groups of sites and when the species occurs in all sites of those groups. Indicator species analyses were run using the package “indicpecies” (version 1.7.6) in R^75^. In the present study, the IndVal values were separately obtained according to the five taxonomic groups.

Considering that dominant taxa would serve as an important trophic mediator in food webs, we identified the dominant taxa of individual taxonomic groups (Supplementary Table 3) to characterize the most important trophic relations in each cluster using the following formula^40^:

$$D_{ij}^{\prime } = F_{ij} \times D_{ij} \times 100 = \left[ {\left( {P_{ij} /P_{j} } \right) \times 100} \right] \times \left[ {\left( {\mathop \sum \limits_{k = 1}^{{P_{j} }} (N_{ik} /N_{k} )} \right)/P_{j} } \right] \times 100$$

where $P_{ij}$ = number of samples including species *i* in cluster *j*; $P_{j}$ = total number of samples in cluster *j*;${ }N_{ik}$ = density of species *i* in the *k*th sample of cluster *j*; and $N_{k}$ = total density of the *k*th sample. All taxa with a frequency ($F_{ij}$) higher than 50% and an abundance higher than the median value of $D_{ij}$ in individual clusters were considered dominant. Then, the trophic interactions between co-occurring dominant taxa in each cluster and/or a combination of clusters were evaluated by employing a stable isotope mixing model.

### Isotope mixing model

Two separate steps were applied to mixing model calculations to delineate pictures of the structure of trophic networks of individual community typologies. First, the relative contributions of putative sources of primary organic matter (riverine SPOM, estuarine and coastal-bay phytoplankton, *P. australis*, microphytobenthos, and *Z. marina*) to the nutrition of primary consumers (suspension feeders and deposit feeders) were evaluated using an IsoSource mixing model^76^. The δ^13^C and δ^15^N values of estuarine and coastal-bay phytoplankton were obtained from site-specific phytoplankton data from stations 1–2 and 5–9^77^. We ran the IsoSource mixing model using the three required input datasets: the mean isotopic values and standard deviations of end-members of organic matter sources, the isotopic values of primary consumer species, and the average trophic enrichment factors (TEFs) for aquatic consumers. In this mixing model calculation, the composition of the nutritional source of consumers was estimated at the individual level. Second, we calculated the relative contribution of benthic affinity prey (*f*) to the nutrition of higher-level consumers (i.e., predators) to identify their trophic links with benthic vs. pelagic pathways, as follows:

$$f = \left[ {\left( \delta{^{13} C_{consumer} - \delta^{13} C_{zooplankton} } \right) - TEF} \right]/\left(\delta {^{13} C_{benthic prey} - \delta^{13} C_{zooplankton} } \right)$$

where $\delta^{13} C_{zooplankton}$ and $\delta^{13} C_{benthic prey}$ represent the δ^13^C values of zooplankton and deposit feeders as isotopic baselines of pelagic and benthic pathways, respectively. The previously published TEF values for diet–animal tissue isotopic fractionations were employed: δ^13^C of 1.3 ± 0.3‰ and δ^15^N of 2.2 ± 0.3‰ for primary consumers and δ^15^N of 3.3 ± 0.26‰ for carnivorous species^78^. This estimation incorporates variance in the diet composition of individual consumers and illustrates the variability of trophic structure of different community typologies.

The trophic position (TP) of individual consumers was also estimated using the following formulas^31,79^:

$$\begin{aligned} & TP_{consumer} = \left[ {\left(\delta {^{15} N_{consumer} -\delta^{15} N_{baseline} } \right) / TEF} \right] +\lambda_{baseline} ; \\ &\delta^{15} N_{baseline} = {(}\delta^{15} N_{benthic prey} \times f) + {[}\delta^{15} N_{zooplankton} \times \left( {1 - f} \right)] \\ \end{aligned}$$

where $TP_{consumer}$ and $\delta^{15} N_{consumer}$ represent the TP and δ^15^N value of the tested consumer, respectively; $\delta^{15} N_{baseline}$ and $\lambda_{baseline}$ represent the δ^15^N value and TP (= 2 in this study) of the baseline organism, respectively; and $TEF$ is the trophic enrichment factor (= 3.3 ± 0.26‰).

### Statistical analyses of environmental and isotopic data

Before statistical analyses, all data were checked for normality and homoscedasticity of variance using the Shapiro–Wilk procedure and Levene’s test, respectively. We tested for significant differences in environmental variables among clusters of the plankton community because of recognizable seasonal as well as spatial patterns. Since individual environmental variables did not meet the assumption for parametric tests, a Kruskal–Wallis test was employed to compare the environmental characteristics among clusters, followed by a Mann–Whitney pairwise comparison test. Analysis of variance followed by a post hoc Tukey multiple comparison was employed to test for significant differences in the δ^13^C and δ^15^N values among primary sources of organic matter and among cluster groups of the plankton community. When necessary to meet homoscedasticity, the δ^13^C values of plankton were transformed to 1/square root ($\left| {\text{x}} \right|$). Student’s *t* test was used to evaluate significant differences in isotopic values for benthic baselines between the estuarine channel and the deep bay, and the Mann–Whitney U test was used to evaluate the δ^13^C values between suspension feeders and deposit feeders in the estuarine channel. In the statistical analyses, an alpha of 0.05 was used as the cutoff for significance. All the above-mentioned statistical tests were performed at *P* < 0.05 the using IBM SPSS Statistics (version 21.0, IBM Corp., Armonk, NY).

### Ethics statement

There was no vertebrate or invertebrate species, which of experiment was required for Institutional Animal Care and Use Committees (IACUC) approval. Fishing and delivery of fish to laboratory was conducted by commercial fishermen, all fish were dead in the net when harvested, and there was no protected vertebrate or invertebrate species in our sample; IACUC approval for this sampling method was not required. Sample collection and export in the sampling area were permitted by Ministry of Oceans and Fisheries of Korea.

## Supplementary information

Supplementary file1
