# Supplementary material for: Identifying patterns in the multitrophic community and food-web structure of a low-turbidity temperate estuarine bay
Source: Sci Rep. 2020 Oct 6;10:16637. doi: 10.1038/s41598-020-73628-6 (PMC7538895; doi:10.1038/s41598-020-73628-6)
Supplement: Supplementary file 1 — Supplementary file1 [file 41598_2020_73628_MOESM1_ESM.pdf]

Supplementary Information

Identifying patterns in the multitrophic community and food-web structure of a low-turbidity temperate estuarine bay

Hee Yoon Kang, Changseong Kim, Dongyoung Kim, Young-Jae Lee, Hyun Je Park, Goutam K. Kundu, Young Kyun Kim, Riaz Bibi, Jaebin Jang, Kwang-Hun Lee, Hyun-Woo Kim, Sung-Gyu Yun, Heeyong Kim & Chang-Keun Kang*

*Correspondence to: Kang C.K. School of Earth Sciences and Environmental Engineering, Gwangju Institute of Science & Technology, Gwangju 61005, Republic of Korea. E-mail: ckkang@gist.ac.kr

Supplementary information includes:

Supplementary Methods

Description of the study site.

Laboratory processing and analyses.

Supplementary References

Supplementary Tables

Supplementary Table 1. Frequency and abundance description of individual clusters.

Supplementary Table 2. List of indicator species of each cluster.

Supplementary Table 3. Dominant taxa of each cluster.

Supplementary Table 4. δ^{13}C and δ^{15}N values (‰) of primary sources of organic matter.

Supplementary Table 5. δ^{13}C and δ^{15}N values (‰) of plankton in each cluster.

Supplementary Table 6. Isotope values of dominant benthic primary consumers.

Supplementary Table 7. primary producers to the nutrition of primary consumers.

Supplementary Table 8. Feasible contributions (%) of primary consumers to the diets of dominant motile crustaceans and fish in the estuarine channel.

Supplementary Table 9. Identification of trophic group of co-occurring dominant taxa.

Supplementary Figures

Supplementary Fig. 1. Visualization of spatial distribution patterns on the SOM units.

Supplementary Methods

Description of the study site. Gwangyang Bay is a semi-closed coastal embayment with an area of ca. 145 km^2, which is located on the southern coast of Korea (Supplementary Fig. 1). It is surrounded by highly populated areas and important industrial complexes and supports large commercial fisheries and mariculture activities. Broad intertidal muddy sand flats at the north end of the bay have been reclaimed for the construction of shipping facilities and industrial complexes, and the area has been dredged to support ship navigation since 1982. Because of this increasing development pressure, the bay requires management for economic and ecological sustainability. The Seomjin River has a catchment area of ca. 5×10^3 km^2, consisting of agricultural and forested land, and flows directly into the north end of the bay[1]. The river discharge carries an annual mean of ca. 120 m^3 s^{-1} of freshwater into the bay but displays substantial seasonal variability, from 30–95 m^3 s^{-1} in winter (base flows) to 300–400 m^3 s^{-1} in the summer monsoon season. The bay has a semidiurnal tidal cycle, with maximum tidal ranges of 3.40 and 1.10 m during the spring and neap tides, respectively. The northern Seomjin River estuarine channel has a depth range of 2.4–8.0 m (depending on the spring-neap tides), an average flushing time of freshwater of 7.0 d at both spring and neap tides, and is thus characterized as a shallow and mesotidal estuary[2]. In the present study, three sites were chosen for the upper estuarine, oligohaline-zone sampling stations. Most (ca. 82%) of the Seomjin River flux is discharged towards Yeosu Sound in the south of the bay, which has a water depth of 10–30 m[1]. The deep bay channel is connected to the Seomjin River in the north and the coastal sea (South Sea of Korea) in the south, forming the polyhaline zone of 32–33 psu (the present study). Six sites were chosen for longitudinal sampling of the bay channel. The surface sediment of the main channel habitat varies from sand near the northern river mouth and sandy mud in the mesohaline zone to silty clay in the deep bay channel[3,4].

Longitudinal patterns of macrobenthic assemblages were previously observed[3–5]. The species number and density of polychaetes, the most dominant taxonomic group of the microbenthic community, decrease drastically from the polyhaline zone of the deep bay channel through the mesohaline zone to the oligohaline zone, with an apparent shift in the dominant taxa. The sources and dynamics of dissolved inorganic nutrients[6,7] and the distributions of phytoplankton[8–10], zooplankton[8,11] and fish assemblage[12,13] have seasonal and longitudinal heterogeneities. Furthermore, ecologically diversified habitat types are developed along the main channel within the bay, e.g., a common reed *Phragmites australis* community

colonized in the upper estuarine supralittoral wetland, an intertidal muddy sand flat in the northern part, and eelgrass *Zostera marina* beds along the low tide line on the fringe of the intertidal muddy sand flat. Resuspended benthic microalgae on the intertidal sandflat (an annual mean of 35.3 ± 33.5 mg chlorophyll *a* m^{-2} in the top 5 mm of the sediment)[14] is known to support a considerable part of the nutrition of adjacent subtidal macrobenthic consumers[15]. By contrast, direct and indirect connectivity between the main channel and other habitats is still unresolved. The above description shows the highly heterogeneous conditions in the system but represents the initial stage of our knowledge of patterns of ecosystem dynamics.

Laboratory processing and analyses. After transportation to the laboratory, the water for SPM quantification was filtered onto pre-combusted and pre-weighed Whatman glass fibre filters (GF/F; 47 mm, 0.7 µm pore size). The filters were then dried for 48 h at 60°C in a drying oven and reweighed after recovering to room temperature in a desiccator. The total SPM concentration was calculated by weighing the filter before and after filtration of a known volume of water.

The water for the chlorophyll *a* measurement was also filtered onto a pre-combusted Whatman GF/F filter, extracted in 90% acetone for 24 h in the dark at −20°C, and its concentration was determined using a fluorometer (Turner Designs Model 10 AU 005, Sunnyvale, CA, USA) according to Holm-Hansen et al.[16].

Water samples for inorganic nutrient analysis were thawed overnight at low temperature (2°C) and brought to room temperature prior to analysis. Concentrations of phosphate (PO_4), ammonium (NH_4), nitrite (NO_2), nitrate (NO_3), and silicate (SiO_2) were colorimetrically determined using a QuAAtro nutrient analyser (SEAL Analytical GmbH, Norderstedt, Germany). These measurements followed the procedures developed by Murphy and Riley[17] for PO_4, Hansen and Grasshoff[18] for NO_2, NO_3, and SiO_2, and Helder and de Vries[19] for NH_4.

Phytoplankton species were identified and counted in 100–200 µl of the 20 ml concentrate under 200 or 400 magnification using a Nikon's Eclipse E600 microscope (Nikon, Tokyo, Japan).

In the laboratory, the species of mesozooplankton were identified and counted under a Nikon's SMZ 645 stereoscopic microscope (Nikon, Tokyo, Japan) using a Bogorov counting chamber after a resuspension of the sample with a known volume of filtered seawater.

Macrobenthic invertebrates, once sorted in the laboratory, were identified to the species level using a Nikon's SMZ 645 stereoscopic microscope (Nikon, Tokyo, Japan) and then

counted. When necessary, the biomass of organisms was estimated by weighing after drying in an oven for 48 h at 60°C.

Nekton samples were sorted in the laboratory, transferred to 70% ethanol, and identified to the lowest possible taxon. When necessary, a dissecting stereomicroscope was used to identify small individuals to the species level. Once counted, individual fish were measured (mm, standard length) and weighed (wet weight, g) to allow abundance, biomass, and frequency of occurrence analyses.

For defatting zooplankton and fish tissues before isotope analysis, while mathematical correction models were designed to adjust the effects of lipids on $\delta^{13}C$ values based on the tissue C:N ratio[20,21], our $\delta^{13}C$ values of lipid-free tissues varied substantially from the mathematically corrected values[22,23]. To avoid this effect, zooplankton and fish tissue samples were defatted in a mixture (2:1:0.8) of methanol, chloroform and water before isotope analysis[24]. Because such a consistent difference in the $\delta^{13}C$ values was undetectable for other invertebrates whose lipid contents were very low (< 5% of dry tissue weight) compared to those (range 4−34%; > 10% in most cases) in fish[25], the defatting of invertebrate tissues was excluded in the present study.

Supplementary References

1. Kim, B. J., Ro, Y. J., Jung, K. Y. & Park, K. S. Numerical modeling of circulation characteristics in the Kwangyang Bay estuarine system. *J. Korean Soc. Coast. Ocean Eng.* **26**, 253–266 (2014).
2. Shaha, D. C., Cho, Y. K., Kim, T. W. & Valle-Levinson, A. Spatio-Temporal Variation of Flushing Time in the Sumjin River Estuary. *Terr. Atmos. Ocean. Sci.* **23**, 119–130 (2012).
3. Kim, Y. H. & Shin, H. C. A benthic polychaete assemblage off the Korean south coast (Gwangyang Bay and Yeosu Sound). *Fish. Aquat. Sci*. **13**, 157–166 (2010).
4. Kang, S. H., Lee, J. H., Park, S. W. & Shin, H. C. Temporal and spatial distribution of benthic polychaetous communities in Seomjin River Estuary. *The Sea J. Korean Soc. Oceanogr.* **19,** 243–255 (2014).
5. Choi, J. W., Hyun, S. & Chang, M. The summer benthic environmental conditions assessed by the functional groups of macrobenthic fauna in Gwangyang Bay, southern coast of Korea. *Korean J. Environ. Biol*. **21,** 101–113 (2013).
6. Jang, P. K. *et al*., Spatial and temporal distribution of inorganic nutrients and factors

controlling their distributions in Gwangyang Bay. *Ocean Polar Res*. **27**, 359–379 (2005).

7. Lee, Y. S. & Kang, C. K. Causes of COD increases in Gwangyang Bay, South Korea. *J. Environ. Monit.* **12**, 1537–1546 (2010).
8. Kwon, K. Y., Lee, P. K., Park, C., Moon, C. H. & Park, M. O. Biomass and species composition of phytoplankton and zooplankton along the salinity gradients in the Seomjin River estuary. *The Sea J. Korean Soc. Oceanogr*. **6**, 93–102 (2001).
9. Park, M. O. *et al*. The species composition of phytoplankton along the salinity gradients in the Seomjin River estuary in autumn, 2000: comparison of HPLC analysis and microscopic observations. *Algae* **16**, 179–188 (2001).
10. Baek, S. H., Kim, D., Son, M., Yun, S. M. & Kim, Y. O. Seasonal distribution of phytoplankton assemblages and nutrient enriched bioassays as indicators of nutrient limitation of phytoplankton growth in Gwangyang Bay, Korea. *Estuar. Coast. Shelf Sci.* **163**, 265–278 (2015).
11. Jang, M. C., Jang, P. G., Shin, K. S., Park, D. W. & Chang, M. Seasonal variation of zooplankton community in Gwangyang Bay. *Korean J. Environ. Biol*. **22**: 11–29 (2004).
12. Cha, S. S. & Park, K.J. Seasonal changes in species composition of fishes collected with a bottom trawl in Kwangyang Bay, Korea. Korean *J. Ichthyol*. **9**, 235–243 (1997).
13. Kwak, S. N., Huh, S. H. & Kim, H.W. Change in fish assemblage inhabiting around Dae Island in Gwangyang Bay, Korea. *J. Korean Soc. Mar. Environ. Safe*. **18**, 175–184 (2012).
14. Kang, C. K. *et al*. Microphytobenthos seasonality determines growth and reproduction in intertidal bivalves. *Mar. Ecol. Prog. Ser.* **315**, 113–127. (2006)
15. Kang, C. K. *et al*. Trophic importance of benthic microalgae to macrozoobenthos in coastal bay systems in Korea: dual stable C and N isotope analyses. *Mar. Ecol. Prog. Ser.* **259**, 79–92 (2003).
16. Holm-Hansen, O., Lorenzen, C. J., Holmes, R. W. & Strickland, J. D. Fluorometric determination of chlorophyll. *J. Conseil* **30**, 3–15 (1965).
17. Murphy, J. & Riley, J. P. A modified single solution method for the determination of phosphate in natural waters. *Anal. Chim. Acta* **27**, 31–36 (1962).
18. Hansen, H. P. & Grasshoff, K. Automated chemical analysis. In *Methods of Seawater Analysis* (eds. Grasshoff, K., Kremling, K. & Ehrhardt, M.) 347–379 (Verlag Chemie, Weinheim, 1983).
19. Helder, W. & de Vries, R. T. P. An automatic phenol-hypochlorite method for the determination of ammonia in sea and brackish water. *Neth. J. Sea Res*. **13,** 154–160 (1979).

20. Sweeting, C. J., Polunin, N. V. C. & Jennings, S. Effects of chemical lipid extraction and arithmetic lipid correction on stable isotope ratios of fish tissues. *Rapid Commun. Mass Spectrom*. **20**, 595–601 (2006).
21. Smyntek, P. M., Teece, M. A., Schulz, K. L. & Thackeray, S. J. A standard protocol for stable isotope analysis of zooplankton in aquatic food web research using mass balance correction models. *Limnol. Oceanogr*. **52**, 2135–2146 (2007).
22. Logan, J.M. *et al*. Lipid corrections in carbon and nitrogen stable isotope analyses: comparison of chemical extraction and modelling methods. *J. Animal Ecol*. **77**, 838–846 (2008).
23. Mintenbeck, K., Brey, T., Jacob, U., Knust, R. & Struck, U. How to account for the lipid effect on carbon stable-isotope ratio ($\delta^{13}C$): sample treatment effects and model bias. *J. Fish Biol.* **72**, 815–830 (2008).
24. Bligh, E. G. & Dyer, W. J. A rapid method of total lipid extraction and purification. *Can. J. Biochem. Physiol*. **37**, 911–917 (1959).
25. National Fisheries Research and Development Institute (NFRDI). Chemical Composition of Marine Products in Korea. (NFRDI, Busan, Korea, 2009).
Available: http://portal.nfrdi.re.kr/page?id=aq_seafood_1.

Supplementary Tables

Supplementary Table 1 | Frequency and abundance description of individual clusters. Frequency (*F*, the number of samples) and log-transformed [ln(abundance + 1)] abundances (*A*, median and range) of taxa accounting for at least 5% of the total abundances of plankton, macrobenthos, and nekton assemblages in each cluster. MAWC, median abundances for whole clusters. Abundance units are Cells l^{-1} for phytoplankton; Individuals m^{-3} for zooplankton; Individuals m^{-2} for macrobenthos; and Individuals 0.03 km^{-2} for nekton.

	Cluster A		Cluster B		Cluster C		Cluster D		Cluster E		
	F	*A*	*F*	*A*	*F*	*A*	*F*	*A*	*F*	*A*	MAWC
**Phytoplankton**											
All taxa	8	7.72 / 4.62–13.93	24	7.47 / 4.62–12.92	7	8.44 / 5.53–12.72	7	7.93 / 4.62–12.40	8	8.38 / 5.53–12.97	
Taxon											
Cerataulina pelagica	4	8.54 / 6.91–9.85	5	5.71 / 5.53–7.24	2	5.87 / 5.53–6.22	2	6.45 / 6.22–6.69	4	6.22 / 5.53–7.60	6.62
Chaetoceros curvisetus	4	8.35 / 7.47–9.56	19	7.72 / 6.62–10.20	7	10.78 / 9.76–11.12	7	8.29 / 7.50–9.30	7	9.02 / 7.47–9.84	8.76
Chaetoceros debilis			1	8.01 /							8.01
Chaetoceros decipiens			2	8.94 / 8.41–9.47			4	6.40 / 5.71–8.85	5	8.01 / 7.31–8.89	8.01
Chaetoceros spp.	5	8.09 / 7.72–8.70	14	7.68 / 6.62–10.02	7	9.19 / 8.16–9.45	7	9.66 / 9.07–10.43	7	9.16 / 8.23–9.88	8.79
Cylindrotheca closterium	4	8.00 / 7.13–9.47	21	7.72 / 5.53–11.19	7	8.74 / 8.09–8.85	1	7.72 /	3	7.31 / 5.53–8.09	7.82
Cymbella spp.	8	7.14 / 4.62–8.23	11	6.22 / 4.62–9.02	5	5.53 / 5.53–6.22	2	5.53 / 5.53–5.53	1	5.53 /	6.22
Diatoma sp.	8	7.39 / 4.62–8.66	6	6.42 / 5.53–10.34	1	5.53 /					6.69
Eucampia zodiacus	3	10.24 / 5.53–11.35	5	8.52 / 4.62–12.92	1	8.47 /	2	9.00 / 6.22–11.78	6	9.24 / 5.53–12.68	8.52
Eutreptiella spp.	5	9.09 / 6.22–10.55	18	6.77 / 5.53–9.13	7	7.31 / 5.53–8.35	4	5.76 / 4.62–7.13	4	6.42 / 6.22–7.60	6.77
Fragilaria sp.	1	9.47 /	1	7.92 /							8.70
Fragilariopsis capucina	2	8.64 /7.72–9.56	1	8.09 /					1	6.62 /	7.90
Gonyaulax sp.	5	8.04 / 5.53–10.17	10	6.22 / 5.53–7.47	1	5.53 / 5.53–5.53	7	7.82 / 4.62–8.90	7	8.47 / 5.53–11.06	6.80
Guinardia delicatula	3	5.30 / 5.30–5.53	4	6.56 / 5.53–8.41	6	8.40 / 6.91–9.62	6	6.31 / 4.62–7.47	6	7.37 / 5.53–8.89	6.91
Melosira varians	2	7.19 / 6.91–7.47	4	7.31 / 6.22–8.35	1	5.53 /					7.31
Nanoflagellates	6	9.53 / 8.13–12.50	22	10.08 / 5.30–12.53	7	11.78 / 10.22–11.93	7	8.41 / 5.99–9.95	8	9.04 / 7.72–9.53	9.49
Nitzschia sp. (< 20 μm)	5	6.62 / 5.30–7.60	20	7.22 / 5.30–9.08	7	7.82 / 7.13–8.52	1	5.53 /	1	5.53 /	7.13
Nitzschia sp. (> 20 μm)	6	7.86 / 5.71–9.51	13	6.62 / 4.62–10.28	6	6.62 / 6.22–8.16			2	7.26 / 6.91–7.60	6.91
Prorocentrum triestinum	2	5.87 / 5.53–6.22	2	6.56 / 6.22–6.91			1	8.99 /	5	11.37 / 10.53–12.97	9.76
Pseudo-nitzschia spp.	8	7.62 / 4.62–10.65	22	7.82 / 5.53–11.43	7	8.01 / 6.91–8.85	7	9.91 / 7.13–11.76	8	9.25 / 7.13–12.21	8.12
Rhizosolenia setigera	4	6.26 / 5.53–6.91	6	7.82 / 5.53–11.10	2	5.87 / 5.53–6.22	6	11.90 / 7.47–12.40	4	5.87 / 5.53–8.01	7.19
Skeletonema costatum	8	12.43 / 11.76–13.93	21	8.78 / 6.91–11.50	7	11.78 / 10.56–12.72	7	8.70 / 7.31–9.39	6	9.11 / 6.22–10.36	9.31
Thalassionema frauenfeldii	1	5.53 /	7	6.22 / 4.62–8.41			6	5.96 / 4.62–7.31	8	8.19 / 5.53–8.92	6.77
Thalassionema nitzschioides	5	7.47 / 5.53 /8.74	12	6.97 / 5.53–8.92	7	8.74 / 8.35–9.16	3	8.29 / 5.30–10.62	8	10.50 / 8.35–11.43	8.35

Thalassiosira rotula	4	9.58 / 5.53–12.09	8	5.53 / 4.62–10.39	7	7.60 / 5.53–8.01	1	8.85 /	3	7.31 / 6.22–7.72	7.31
Thalassiosira spp. (< 40 μm)	6	6.69 / 5.53–8.57	21	7.13 / 5.53–8.48	7	9.05 / 8.57–9.33	7	7.82 / 7.09–8.61	8	8.16 / 6.22–8.57	7.31
**Zooplankton**											
All taxa	8	2.78 / 0.20–7.59	24	2.35 / 0.27–7.37	7	3.64 / 0.85–8.77	7	4.11 /1.35–9.18	8	4.08 / 0.94–8.61	
Taxon											
Acartia hongi	1	2.66 /	1	3.55 /	1	1.25 /	1	3.48 /	5	3.81 / 1.42–4.98	3.11
Acartia hudsonica	3	5.25 / 1.12–6.19	1	3.42 /							4.33
Acartia ohtsukai	3	2.39 / 0.20–4.19	16	1.55 / 0.58–5.26			6	5.55 / 1.90–6.11			2.47
Acartia omorii	5	3.09 / 0.50–5.74	18	1.46 / 0.33–6.34	7	4.95 / 3.38–5.41	6	2.62 / 2.32–7.25	8	4.08 / 2.63–6.60	2.74
Calanus sinicus	2	1.29 / 0.71–1.88	14	2.70 / 0.38–4.80	7	3.54 / 1.79–4.81	1	3.61 /	2	3.52 / 3.33–3.70	2.82
Centropages abdominalis	2	2.57 / 0.93–4.21	5	1.13 / 0.35–6.48	7	4.05 / 2.91–5.11	1	5.62 /	3	5.56 / 5.43–6.30	4.09
Cirriped larvae	5	2.73 / 0.63–5.60	14	2.54 / 0.45–5.03	7	5.22 / 4.61–5.96	6	4.65 / 2.95–5.39	6	5.14 / 1.89–7.00	4.36
Copepodite of *Acartia*	1	2.10 /	5	0.65 / 0.27–1.77	7	3.30 / 1.74–3.69					2.10
Copepodite of *Centropages*			3	0.60 / 0.58–1.23							0.60
Corycaeus affinis	3	1.88 / 0.71–3.04	19	2.88 / 0.87–4.43	7	2.97 / 1.61–4.09	4	3.08 / 1.55–4.82	8	4.48 / 2.82–4.98	3.09
Decapod larvae	2	3.42 / 3.39–3.46	14	1.62 / 1.01–5.38	3	1.31 / 1.25–1.55	6	4.89 / 3.80–5.19	6	3.86 / 1.74–5.43	2.91
Doliolum sp.			1	6.30 /			4	4.58 / 3.63–6.12			4.93
Eurytemora pacifica	3	2.91 / 2.47–3.82	1	1.93 /	1	1.25 /					2.47
Evadne nordmanni									4	3.66 / 2.60–5.46	3.66
Evadne sp.	2	0.62 / 0.20–1.03	2	4.28 / 1.65–6.92			5	5.69 / 1.53–7.01			3.01
Fish egg	1	1.25 /	3	1.13 / 0.81–1.23			1	1.90 /	5	4.98 / 3.36–5.77	2.63
Harpacticoids unid.	4	3.58 / 2.22–5.87	5	2.20 / 0.63–2.55	2	1.82 / 1.07–2.57	2	3.32 / 2.52–4.11	7	3.58 / 2.26–4.72	2.58
Hydromesae unid.	2	4.85 / 3.84–5.87	15	1.85 / 0.63–4.49	4	1.53 / 1.25–1.79	7	3.81 / 2.52–5.26	4	3.08 / 2.26–3.65	2.29
Noctiluca scintillans	3	3.57 / 2.73–6.70	19	4.73 / 1.23–7.37	7	8.29 / 8.15–8.77	7	8.97 / 7.69–9.18	8	8.30 / 7.26–8.61	7.31
Oikopleura dioica	2	1.78 / 0.41–3.14	9	2.08 / 1.01–4.94	7	3.15 / 2.67–4.42	2	3.27 / 2.26–4.29	8	2.80 / 1.64–5.25	2.77
Oithona spp.	3	2.39 / 2.13–3.54	8	1.69 / 0.33–5.82	2	1.03 / 0.85–1.21	1	5.26 /	4	4.68 / 0.94–4.86	1.93
Ophiopluteus larvae	1	1.79 /	9	2.11 / 0.87–4.29	6	1.93 / 1.05–3.04	2	2.54 / 2.52–2.56	7	2.51 / 1.36–4.28	2.51
Paracalanus parvus	7	3.04 / 0.50–5.27	22	3.32 / 0.87–6.85	7	5.36 / 5.11–5.82	7	3.64 / 2.20–6.60	8	2.53 / 1.24–6.54	3.68
Pseudodiaptomus koreanus	5	4.39 / 1.25–7.59	4	2.66 / 1.54–3.55			4	2.85 / 1.55–4.20	1	2.46 /	2.85
Sagitta crassa	2	1.51 / 0.41–2.60	18	3.29 / 1.88–5.37	7	3.85 / 3.30–5.15	2	3.73 / 3.22–4.24	6	4.24 / 2.05–4.74	3.68
Sagitta sp.	1	1.03 /	6	3.09 / 2.39–3.57			5	3.54 / 2.70–4.08			3.09
Sinocalanus tenellus	5	4.10 / 2.39–5.65	5	2.29 / 1.79–4.85			1	1.35 /			2.48
Tortanus dextrilobatus	4	3.62 / 0.93–4.77	4	1.78 / 0.53–2.61			1	2.11 /			2.11
	**Cluster 1**		**Cluster 2**		**Cluster 3a**		**Cluster 3b**		**Cluster 3c**		
	F	*A*	*F*	*A*	*F*	*A*	*F*	*A*	*F*	*A*	MAWC
**Macrobenthos**											
All taxa	7	2.35 / 0.69–5.46	4	2.71 / 0.69–5.59	27	1.10 / 0.69–4.51	10	1.61 / 0.69–4.26	6	1.61 / 0.69–5.30	

Taxon											
Aonides oxycephala	1	0.69 /			8	1.67 / 0.69–2.20			2	1.04 / 0.69–1.39	1.39
Aricidea (*Allia*) *elongata*					13	0.69 / 0.69–1.10	7	0.69 / 0.69–2.30	3	0.69 / 0.69–1.61	0.69
Capitella capitata	2	0.90 / 0.69–1.10	4	2.38 / 0.69–2.64	25	2.30 / 0.69–3.66	8	2.38 / 1.79–2.94	6	2.39 / 1.39–2.94	2.30
Chaetozone setosa					9	1.10 / 0.69–3.43	4	1.59 / 0.69–2.48	3	1.10 / 0.69–2.77	1.24
Chaetozone sp.	1	0.69 /			8	1.10 / 0.69–1.61	7	1.39 / 0.69–2.20	4	0.69 / 0.69–2.30	1.10
Corbicula japonica	7	4.80 / 4.16–5.46	4	2.92 / 2.71–3.40	1	0.69 /					4.31
Corophium japonica					6	1.70 / 0.69–2.08	4	1.52 / 0.69–2.56	2	1.67 / 0.69–2.64	1.70
Diastylis rugosa	1	0.69 /			6	1.24 / 0.69–2.48					1.10
Glycera alba	1	1.10 /			9	0.69 / 0.69–1.79	5	1.95 / 0.69–2.30	2	1.24 / 1.10–1.39	1.10
Glycera chirori			1	0.69 / 0.69–0.69	16	1.24 / 0.69–2.56	8	1.39 / 0.69–2.71	5	1.39 / 0.69–1.79	1.39
Magelona japonica	1	1.10 /			15	1.61 / 0.69–2.89	8	1.79 / 1.10–3.50	6	4.38 / 3.50–5.30	1.94
Melita sp.					8	0.90 / 0.69–2.77	8	2.82 / 1.10–4.14	4	2.44 / 1.39–3.58	1.95
Musculus senhausia	2	0.90 / 0.69–1.10	1	1.95 / 1.95–1.95	5	1.39 / 0.69–4.51					1.24
Neanthes japonica	6	3.86 / 2.40–5.23									3.86
Paraprionospio pinnata					12	1.24 / 1.10–2.08	8	1.24 / 0.69–2.20	2	1.50 / 1.39–1.61	1.39
Philine argentata					7	0.69 / 0.69–1.61	4	1.24 / 0.69–1.61	2	0.69 / 0.69–0.69	0.69
Praxillella affinis	1	1.10 /			6	1.39 / 0.69–2.08	6	1.70 / 1.10–3.30	4	1.70 / 0.69–1.79	1.61
Praxillella gracillis					4	1.24 / 0.69–2.40	4	2.28 / 1.39–3.81	1	0.69 /	1.39
Prionospio japonicus	7	2.30 / 0.69–4.79	4	4.26 / 3.78–5.59	7	1.10 / 0.69–3.83					2.25
Scoletoma longifolia					10	2.17 / 0.69–3.00	7	3.50 / 1.39–4.26	5	2.20 / 2.08–3.56	2.52
Sigambra tentaculata	1	0.69 /			19	1.39 / 0.69–2.48	8	1.24 / 0.69–2.08	4	1.10 / 0.69–2.20	1.39
Sternaspis scutata					16	1.10 / 0.69–2.56	9	1.39 / 0.69–1.95	6	1.24 / 0.69–2.40	1.10
Tharyx monilaris	1	1.10 /			11	1.10 / 0.69–2.48	5	2.89 / 0.69–3.76	1	3.71 /	1.24
Theora fragilis					6	0.69 / 0.69–1.79	4	1.89 / 1.10–3.40	1	1.10 /	1.10
Xenophthalmus pinnotheroides			1	0.69 /	2	0.90 / 0.69–1.10	7	2.56 / 0.69–3.66	2	1.32 / 0.69–1.95	1.24

	**Cluster I**		**Cluster II**		**Cluster III**		**Cluster IV**		
	F	*A*	*F*	*A*	*F*	*A*	*F*	*A*	MAWC
**Nekton**									
**Crustaceans**									
All taxa	9	1.50 / 0.69–4.76	4	2.56 / 1.10–6.11	3	2.40 / 0.69–7.49	2	4.29 / 0.69–5.93	
Taxon									
Eucrate crenata	1	0.69 /	2	1.99 / 1.10–2.89			1	2.20 / 2.20–2.20	1.65
Charybdis japonica	6	0.69 / 0.69–1.79	3	1.61 / 1.39–2.71	1	0.69 /	2	2.32 / 1.39–3.26	1.24
Oratosquilla oratoria	3	2.56 / 2.20–4.76	4	4.12 / 3.40–5.12	3	3.09 / 3.00–3.64	2	5.79 / 5.70–5.89	3.58
Palaemon gravieri			1	1.10 /	3	2.64 / 1.61–5.70	1	4.41 / 4.41–4.41	2.64
Alpheus japonicus	4	0.69 / 0.69–1.79	2	2.09 / 1.61–2.56	3	1.95 / 1.79–2.40	2	3.52 / 2.30–4.74	1.79
Charybdis bimaculata	3	2.40 / 1.10–2.56	4	3.17 / 1.79–6.11	3	2.48 / 1.39–2.56	2	5.51 / 5.38–5.64	2.56
Crangon hakodatei	4	2.41 / 1.61–4.36			3	6.58 / 5.89–7.49	2	4.27 / 3.85–4.69	4.36

Parapenaeopsis tenella	3	1.79 / 1.10–2.77	4	3.00 / 1.10–3.69	3	3.18 / 2.40–3.61	2	4.63 / 4.17–5.09	2.98
Metapenaeus joyneri	1	4.20 /	3	3.22 / 2.48–5.12	3	1.10 / 0.69–1.95	2	3.66 / 1.39–5.93	2.48
Alpheus digitalis	2	1.24 / 1.10–1.39	2	1.39 / 1.39–1.39	1	2.08 /	2	3.60 / 3.37–3.83	1.39
Trachysalambria curvirostris	4	1.70 / 1.39–2.20	3	1.61 / 1.61–3.93	1	1.79 /	1	4.49 /	1.79
Portunus trituberculatus	3	0.69 / 0.69–0.69	3	1.95 / 1.61–2.48	1	0.69 /	1	0.69 /	0.69
**Cephalopods**									
All taxa	9	1.79 / 0.69–3.37	4	1.61 / 0.69–4.44	3	1.95 / 0.69–3.78	2	2.83 / 1.39–6.20	
Taxon									
Euprymna morsei	1	1.10 /			3	1.95 / 1.10–2.71			1.52
Octopus variabilis	2	1.04 / 0.69–1.39	4	1.39 / 0.69–1.61	2	0.69 / 0.69–0.69	2	3.03 / 2.77–3.30	1.39
Octopus membranaceus	1	2.30 /	2	1.35 / 1.10–1.61	3	1.39 / 1.10–2.56	2	2.25 / 1.61–2.89	1.61
Loligo japonica	3	2.08 / 1.95–3.37	4	4.06 / 3.81–4.44	3	3.74 / 3.18–3.78	2	6.14 / 6.07–6.20	3.80
Sepia esculenta	2	1.70 / 1.61–1.79	3	1.61 / 1.39–2.08			2	1.98 / 1.39–2.56	1.61
**Fish**									
All taxa	9	1.50 / 0.69–7.38	4	3.48 / 1.10–5.92	3	2.14 / 0.69–7.31	2	3.55 / 0.69–8.89	
Taxon									
Pleuronectes yokohamae	6	1.10 / 0.69–1.39	1	1.10 /	2	1.39 / 0.69–2.08	2	1.87 / 0.69–3.04	1.10
Pholis nebulosa	3	1.39 / 0.69–1.39			3	0.69 / 0.69–2.77	2	1.70 / 0.69–2.71	1.04
Tribolodon hakonensis	4	1.24 / 0.69–2.40							1.24
Amblychaeturichthys hexanema	4	0.90 / 0.69–3.33	4	3.12 / 2.30–3.91	3	3.09 / 1.10–4.17	2	2.43 / 2.30–2.56	2.56
Setipinna tenuifilis	2	3.61 / 2.64–4.58			1	7.31 / 7.31–7.31	1	2.94 /	3.76
Konosirus punctatus	5	0.69 / 0.69–3.40	1	3.53 /	2	3.35 / 1.10–5.59			1.45
Hexagrammos otakii	3	1.10 / 0.69–2.56			2	0.69 / 0.69–0.69	2	2.11 / 1.39–2.83	1.10
Cynoglossus joyneri	4	2.42 / 1.61–3.56	4	3.31 / 2.77–3.93	3	2.56 / 2.30–3.14	2	4.00 / 3.85–4.16	3.04
Okamejei kenojei	3	2.77 / 2.20–3.37	4	2.67 / 1.10–2.94	3	2.56 / 1.10–2.94	2	3.93 / 3.50–4.37	2.74
Ctenotrypauchen microcephalus	2	1.59 / 1.39–1.79	2	1.98 / 1.39–2.56	2	2.69 / 2.20–3.18	1	1.61 /	1.79
Takifugu poecilonotus	4	1.10 / 0.69–1.39							1.10
Leiognathus nuchalis	5	3.93 / 2.94–7.38	4	5.35 / 3.33–5.51			2	7.59 / 6.29–8.89	5.49
Johnius grypotus	1	5.00 /	2	4.28 / 3.37–5.20	2	1.52 / 1.10–1.95	2	4.17 / 4.04–4.29	4.04
Pennahia argentata	2	4.04 / 3.47–4.62	4	4.57 / 3.43–5.00	1	0.69 /	2	5.20 / 4.79–5.62	4.62
Thryssa kammalensis	2	2.38 / 1.39–3.37	4	4.83 / 1.61–5.92			2	5.30 / 3.61–6.99	4.00

Supplementary Table 2 | List of indicator species of each cluster. For each species, the association with clusters is provided by its relative abundance (***A***) in a cluster over all clusters, relative frequency of occurrence (***B***) in that cluster, and square root of the IndVal index (IndVal.g) with significance (*P*-value). Significant IndVal values represent the contribution of each indicator species to one or more clusters on the self-organizing map and characterize the IndVal groups. Values were generated using R software indicpecies version 1.7.6 (R Core Team (2016). R: A language and environment for statistical computing. R Foundation for Statistical Computing, Vienna, Austria. http://www.R-project.org/).

					**Cluster**				
Taxon	***A***	***B***	IndVal.g	*P*	A	B	C	D	E
**Phytoplankton**									
Diatoma sp.	0.52	1.00	0.72	0.014	+				
Nitzschia sp. (< 20um)	0.99	0.82	0.90	< 0.001	+	+	+		
Nitzschia sp. (> 20um)	0.95	0.64	0.78	0.029	+	+	+		
Cylindrotheca closterium	0.93	0.82	0.88	0.006	+	+	+		
Cymbella spp.	0.96	0.62	0.77	0.029	+	+	+		
Thalassiosira rotula	0.93	0.73	0.83	0.022	+		+		
Skeletonema costatum	0.96	1.00	0.98	< 0.001	+		+	+	
Gonyaulax sp.	0.98	0.83	0.90	< 0.001	+			+	+
Chaetoceros curvisetus	0.95	0.87	0.91	0.027		+	+	+	+
Chaetoceros spp.	0.88	0.96	0.92	< 0.001			+	+	+
Guinardia delicatula	0.96	0.82	0.89	0.002			+	+	+
Thalassionema nitzschioides	0.85	1.00	0.92	< 0.001			+		+
Rhizosolenia setigera	0.96	0.86	0.91	< 0.001				+	
Thalassionema frauenfeldii	0.91	0.93	0.92	< 0.001				+	+
Chaetoceros decipiens	0.78	0.60	0.68	0.010				+	+
Prorocentrum triestinum	0.99	0.63	0.79	0.003					+
**Zooplankton**									
Sinocalanus tenellus	0.89	0.63	0.75	0.005	+				
Tortanus dextrilobatus	0.93	0.50	0.68	0.011	+				
Pseudodiaptomus koreanus	0.99	0.60	0.77	0.008	+			+	
Harpacticoids unid.	0.87	0.69	0.77	0.007	+				+
Sagitta crassa	0.92	0.80	0.86	0.004		+	+		+
Corycaeus affinis	0.98	0.83	0.90	0.009		+	+	+	+
Acartia sp. (copepodite)	0.95	1.00	0.98	< 0.001			+		
Calanus sinicus	0.64	1.00	0.80	0.005			+		
Oikopleura dioica	0.76	1.00	0.87	< 0.001			+		+
Ophiopluteus larvae	0.70	0.87	0.78	0.011			+		+
Noctiluca scintillans	0.98	1.00	0.99	< 0.001			+	+	+
Cirriped larvae	0.90	0.86	0.88	0.027			+	+	+
Acartia ohtsukai	0.88	0.86	0.89	0.002				+	
Evadne sp.	0.88	0.71	0.79	< 0.001				+	
Sagitta sp.	0.82	0.71	0.76	< 0.001				+	
Doliolum sp.	0.82	0.57	0.68	0.003				+	
Decapod larvae	0.87	0.80	0.84	0.007				+	+
Acartia hongi	0.82	0.63	0.72	< 0.001					+
Evadne nordmanni	1.00	0.50	0.71	< 0.001					+
Fish egg	0.99	0.63	0.79	< 0.001					+

					**Cluster**				
	A	*B*	IndVal	*P*	1	2	3a	3b	3c
**Macrobenthos**									
**Bivalves**									

Corbicula japonica	1.00	1.00	1.00	< 0.001	+	+			
**Crustaceans**									
Xenophthalmus pinnotheroides	0.87	0.70	0.78	0.006				+	
Melita sp.	0.96	0.75	0.85	< 0.001				+	+
**Polychaetes**									
Neanthes japonica	1.00	0.86	0.93	< 0.001	+				
Prionospio japonicus	0.98	1.00	0.99	< 0.001	+	+			
Capitella capitata	0.99	0.92	0.95	< 0.001		+	+	+	+
Sigambra tentaculata	0.98	0.72	0.84	< 0.001			+	+	+
Glycera chirori	0.97	0.67	0.81	0.009			+	+	+
Aricidea elongata	1.00	0.54	0.73	0.031			+	+	+
Paraprionospio pinnata	1.00	0.51	0.72	0.037			+	+	+
Sternaspis scutata	0.78	0.94	0.86	< 0.001				+	+
Scoletoma longifolia	0.92	0.75	0.83	0.002				+	+
Chaetozone unid.	0.86	0.69	0.77	0.006				+	+
Praxillella affinis	0.88	0.63	0.74	0.008				+	+
Magelona japonica	0.88	1.00	0.94	< 0.001					+

					Cluster			
	A	*B*	IndVal	*P*	I	II	III	IV
**Nekton**								
**Crustaceans**								
Charybdis bimaculata	0.99	1.00	1.00	< 0.001		+	+	+
Parapenaeopsis tenella	0.99	1.00	0.99	0.002		+	+	+
Oratosquilla oratoria	0.97	1.00	0.98	0.003		+	+	+
Metapenaeus joyneri	0.97	0.89	0.93	0.046		+	+	+
Crangon hakodatei	0.99	1.00	0.99	0.002			+	+
Alpheus japonicus	0.93	1.00	0.97	0.014			+	+
Palaemon gravieri	1.00	0.80	0.89	0.036			+	+
Alpheus digitalis	0.89	1.00	0.95	0.017				+
**Cephalopods**								
Octopus variabilis	0.95	1.00	0.98	< 0.001		+		+
Sepia esculenta	0.92	0.83	0.87	0.015		+		+
Loligo japonica	0.99	1.00	1.00	< 0.001		+	+	+
Euprymna morsei	0.97	1.00	0.99	0.003			+	
Octopus membranaceus	0.87	1.00	0.93	0.002			+	+
**Fish**								
Thryssa kammalensis	1.00	1.00	1.00	0.002		+		+
Pennahia argentata	0.95	1.00	0.98	< 0.001		+		+
Leiognathus nuchalis	0.93	1.00	0.96	0.02		+		+
Amblychaeturichthys hexanema	0.95	1.00	0.98	0.006		+	+	+
Cynoglossus joyneri	0.94	1.00	0.97	0.003		+	+	+
Okamejei kenojei	0.93	1.00	0.97	0.004		+	+	+
Pholis nebulosa	0.94	1.00	0.97	0.004			+	+
Hexagrammos otakii	0.80	1.00	0.90	0.033				+

Supplementary Table 3 | Dominant taxa in each cluster. Data are median values of log-transformed abundances of dominant taxa in each cluster. Nanoflagellates are mixotrophic but classified into phytoplankton groups in this study. Abundance units are Cells l^{-1} for phytoplankton; Individuals m^{-3} for zooplankton; Individuals m^{-2} for macrobenthos; and Individuals 0.03 km^{-2} for nekton.

	**Cluster**				
	A	B	C	D	E
**Phytoplankton**					
Cerataulina pelagica	8.54				
Cymbella spp.	7.14				
Diatoma sp.	7.39				
Eutreptiella spp.	9.09	6.77			
Nitzschia sp. (> 20 μm)	7.86	6.62			
Gonyaulax sp.	8.04			7.82	8.47
Chaetoceros spp.	8.09	7.68	9.19	9.66	9.16
Pseudo-nitzschia spp.	7.62	7.82	8.01	9.91	9.25
Skeletonema costatum	12.43	8.78	11.78	8.70	9.11
Thalassiosira spp. (< 40 μm)	6.69	7.13	9.05	7.82	8.16
Chaetoceros curvisetus		7.72	10.78	8.29	9.02
Thalassionema nitzschioides		6.97	8.74		10.50
Cylindrotheca closterium		7.72	8.74		
Nitzschia sp. (< 20 mm)		7.22	7.82		
Guinardia delicatula			8.40	6.31	
Rhizosolenia setigera				11.90	
Thalassionema frauenfeldii				5.96	8.19
Eucampia zodiacus					9.24
Prorocentrum triestinum					11.37
Nanoflagellates	9.53	10.08	11.78	8.41	9.04
**Zooplankton**					
Sinocalanus tenellus	4.10				
Tortanus dextrilobatus	3.62				
Pseudodiaptomus koreanus	4.39			2.85	
Harpacticoids unid.	3.58				3.58
Acartia omorii	3.09	1.46	4.95	2.62	4.08
Paraclanus parvus	3.04	3.32	1.93	3.64	2.53
Cirriped larvae	2.73	2.54	5.22	4.65	5.14
Noctiluca scintillans		4.73	8.29	8.97	8.30
Sagitta crassa		3.29	3.85		4.24
Calanus sinicus		2.70	3.54		
Acartia ohtsukai		1.55		5.55	
Decapod larvae		1.62		4.89	
Hydromesae unid.		1.85		3.81	
Corycaeus affinis		2.88			3.86
Centropages abdominalis			4.05		
Copepodite of *Acartia*			3.30		
Oikopleura dioica			3.15		2.80
Evadne sp.				5.69	
Sagitta sp.				3.54	
Fish egg					4.98

	**Cluster**				
	1	2	3a	3b	3c
**Macrobenthos**					
**Bivalves**					
Corbicula japonica	4.80	2.92			

**Crustceans**					
Xenophthalmus pinnotheroides				2.56	
Melita sp.				2.82	2.44
**Polychaetes**					
Neanthes japonica	3.86				
Prionospio japonicus	2.30	4.26			
Capitella capitata		2.38	2.30	2.38	2.39
Glycera chirori			1.24	1.39	1.39
Magelona japonica			1.61	1.79	4.38
Sigambra tentaculata			1.39	1.24	1.10
Sternaspis scutata			1.10	1.39	1.24
Scoletoma longifolia				3.50	2.20
Paraprionospio pinnata				1.24	
Chaetozone sp.					0.69
Praxillella affinis					1.70

	**Cluster**			
	I	II	III	IV
**Nekton**				
**Crustceans**				
Charybdis japonica	0.69			
Crangon hakodatei	2.41		6.58	4.27
Oratosquilla oratoria		4.12	3.09	5.79
Charybdis bimaculata		3.17	2.48	5.51
Parapenaeopsis tenella		3.00	3.18	4.63
Metapenaeus joyneri		3.22		3.66
Trachysalambria curvirostris	1.70	1.61		
Portunus trituberculatus		1.95		
Palaemon gravieri			2.64	
Alpheus japonicus			1.95	
Alpheus digitalis				3.60
**Cepahalopods**				
Loligo japonica		4.06	3.74	6.14
Octopus variabilis		1.39		
Euprymna morsei			1.95	
**Fish**				
Pleuronectes yokohamae	1.10			
Leiognathus nuchalis	3.93	5.35		7.59
Konosirus punctatus	0.69		3.35	
Cynoglossus joyneri		3.31	2.56	4.00
Amblychaeturichthys hexanema		3.12	3.09	
Pennahia argentata		4.57		5.20
Thryssa kammalensis		4.83		5.30
Okamejei kenojei			2.56	3.93
Ctenotrypauchen microcephalus			2.69	
Johnius grypotus				4.17

Supplementary Table 4 | δ^{13}C and δ^{15}N values (‰) of primary sources of organic matter. Data are the mean ± 1SD. *N* is in parentheses. SPOM represents suspended particulate organic matter. Two-way analysis of variance (ANOVA) was used to test the significance of differences among seasons as well as sources. To meet homoscedasticity, the δ^{13}C values were transformed to $1/\sqrt{|x|}$. *P* indicates the significance of the test. The same superscript in the 'Mean ± SD' column indicates that the mean values are not significantly different (*P* > 0.05, Tukey HSD test).

	δ^{13}C			δ^{15}N		
	df	*F*	*P*	df	*F*	*P*
Source	3	554.792	< 0.001	3	37.302	< 0.001
Season	3	0.724	0.544	3	2.590	0.066
Source × Season	8	2.131	0.055	8	0.097	0.999
Residual	40			40		
Total	55			55		

	February	May	August	November	Mean ± SD
**δ^{13}C**					
Riverine SPOM	—	−24.9 ± 0.1 (2)	−26.9 ± 1.3 (2)	−25.2 ± 0.8 (2)	−25.7 ± 1.2^{b} (6)
Phragmites australis	−27.4 ± 0.5 (4)	−27.7 ± 0.6 (4)	−27.2 ± 0.4 (4)	−27.5 ± 0.8 (4)	−27.5 ± 0.6^{a} (16)
Microphytobenthos	−15.4 ± 0.6 (4)	−14.8 ± 1.0 (4)	−14.3 ± 1.0 (5)	−14.9 ± 0.5 (4)	−14.8 ± 0.8^{c} (17)
Zostera marina	−8.0 ± 2.0 (3)	−7.9 ± 1.6 (4)	−8.4 ± 0.5 (4)	−8.9 ± 1.3 (3)	−8.6 ± 1.0^{d} (15)
**δ^{15}N**					
Riverine SPOM	—	5.4 ± 0.6 (2)	5.6 ± 0.3 (2)	5.2 ± 0.4 (2)	5.4 ± 0.4^{e} (6)
Phragmites australis	5.7 ± 0.6 (4)	6.3 ± 0.5 (4)	6.5 ± 0.5 (4)	6.1 ± 0.4 (4)	6.2 ± 0.6^{e} (16)
Microphytobenthos	8.0 ± 0.8 (4)	8.4 ± 0.8 (4)	8.8 ± 0.8 (5)	8.5 ± 1.0 (4)	8.4 ± 0.8^{f} (17)
Zostera marina	7.6 ± 1.4 (6)	8.1 ± 0.6 (3)	8.7 ± 0.5 (3)	8.6 ± 0.8 (3)	8.1 ± 1.0^{f} (15)

Supplementary Table 5 | δ^{13}C and δ^{15}N values (‰) of plankton in each cluster. Data are the mean ± 1SD. *N* is in parentheses. Analysis of variance (ANOVA) was used to test the significance of differences among clusters. *P* indicates the significance of the test. To meet homoscedasticity, the δ^{13}C values were transformed to $1/\sqrt{|x|}$. The same superscript in each column indicates that the mean values are not significantly different ($P > 0.05$, Tukey HSD test). Baseline values of the pelagic pathway for the isotope-mixing model were calculated as the means of the isotope values of areal plankton-community clusters based on the significance by the ANOVA test.

Taxonomic group	Area	Cluster group	δ^{13}C (‰)	δ^{15}N (‰)	Pelagic baseline values	
					δ^{13}C (‰)	δ^{15}N (‰)
Phytoplankton	Estuarine channel	A	−22.2 ± 3.0^{ab} (8)	7.7 ± 2.3 (8)	−22.9 ± 2.6 (13)	7.8 ± 2.1 (50)
		B	−23.9 ± 1.6^{a} (5)	7.1 ± 2.4 (5)		
	Deep bay	B	−20.4 ± 2.0^{bc} (15)	7.6 ± 2.2 (15)	−19.9 ± 1.8 (37)	
		C	−18.6 ± 1.0^{c} (7)	7.9 ± 0.9 (7)		
		D	−20.6 ± 1.9^{bc} (7)	7.9 ± 2.2 (7)		
		E	−19.4 ± 1.6^{bc} (8)	8.6 ± 2.3 (8)		
	ANOVA	df = 5, 44	$F = 6.153$; $P < 0.001$	$F = 0.403$; $P = 0.844$		
Zooplankton	Estuarine channel	A	−23.1 ± 3.3^{e} (8)	9.5 ± 1.2 (8)	−23.8 ± 2.8 (13)	9.5 ± 1.3 (44)
		B	−25.0 ± 1.1^{e} (5)	10.6 ± 0.8 (5)		
	Deep bay	B	−19.6 ± 1.7^{f} (13)	9.4 ± 1.6 (13)	−19.5 ± 1.2 (31)	
		C	−20.0 ± 0.4^{f} (7)	8.7 ± 0.9 (7)		
		D	−19.0 ± 0.9^{f} (7)	9.8 ± 1.5 (7)		
		E	−18.9 ± 0.5^{f} (4)	9.1 ± 1.1 (4)		
	ANOVA	df = 5, 38	$F = 12.125$; $P < 0.001$	$F = 1.371$; $P = 0.257$		

Supplementary Table 6 | Isotope values of dominant benthic primary consumers. $\delta^{13}C$ and $\delta^{15}N$ values (‰) in each cluster based on the self-organizing map and isotopic baselines of the benthic pathway for the isotope-mixing model. Data are mean ± 1SD; *N* is in parentheses; S, suspension feeder; D, deposit feeder. Benthic baseline values were calculated as areal means of isotope values of deposit feeders in macrobenthos-community clusters. Differences in the $\delta^{13}C$ and $\delta^{15}N$ values between suspension and deposit feeders in the estuarine channel were significant (Mann-Whitney U test, U = 6.5, $P < 0.001$ for $\delta^{13}C$; Student's *t* test, $t_{18} = -3.737$, $P = 0.002$ for $\delta^{15}N$). Differences in the $\delta^{13}C$ and $\delta^{15}N$ values of benthic baselines (i.e., deposit feeders) between the estuarine channel and the deep bay were significant (Student's *t* test, $t_{42} = 16.637$, $P < 0.001$ for $\delta^{13}C$; $t_{42} = -2.990$, $P = 0.005$ for $\delta^{15}N$).

	Estuarine-channel clusters		Deep-bay clusters		
Taxon	1	2	3a	3b	3c
		**$\delta^{13}C$**			
Bivalve					
Corbicula japonica (S)	−24.4 ± 0.5 (6)	−22.1 ± 0.7 (5)			
Crustaceans					
Melita sp. (D)				−16.4 ± 1.3 (3)	
Xenophthalmus pinnotheroides (D)				−17.3 ± 1.2 (4)	
Polychaetes					
Neanthes japonica (D)		−21.2 ± 0.4 (3)			
Prionospio japonicus (D)	−21.3 ± 0.4 (2)	−21.2 ± 0.4(2)			
Capitella capitata (D)		−21.5 ± 1.0(2)	−16.8 ± 0.5(7)		−16.4 ± 0.9 (2)
Magelona japonica (D)				−15.9 ± 0.5 (3)	−16.7 ± 0.4 (5)
Sternaspis scutata (D)				−16.3 ± 1.0 (5)	−16.9 ± 0.2 (3)
Paraprionospio pinnata (D)				−16.8 (1)	
Chaetozone sp. (D)					−17.6 (1)
Praxillella affinis (D)					−16.9 (1)
Benthic baseline values	−21.3 ± 0.6 (9)			−16.7 ± 0.8 (35)	
		**$\delta^{15}N$**			
Bivalve					
Corbicula japonica(S)	10.2 ± 0.4 (6)	10.4 ± 0.9 (5)			
Crustaceans					
Melita sp. (D)				9.6 ± 0.8 (3)	
Xenophthalmus pinnotheroides (D				10.4 ± 0.7 (4)	
Polychaetes					
Neanthes japonica (D)		11.7 ± 0.3 (3)			
Prionospio japonicas (D)	11.5 ± 1.2 (2)	11.5 ± 0.3 (2)			
Capitella capitata (D)		10.9 ± 0.9 (2)	10.7± 0.6 (7)		10.3 ± 0.7 (2)
Magelona japonica (D)				10.2 ± 0.6 (3)	10.8± 0.5 (5)
Sternaspis scutata (D)				10.6 ± 0.9 (5)	9.7 ± 0.9 (3)
Paraprionospio pinnata (D)				12.1 (1)	
Chaetozone sp.(D)					11.8 (1)
Praxillella affinis (D)					11.7 (1)
Benthic baseline values	11.4 ± 0.6 (9)			10.5 ± 0.8 (35)	

Supplementary Table 7 | Feasible contributions (%) of primary producers to the nutrition of primary consumers. Dominant primary producers considered include *Phragmites australis*, locally produced (estuarine vs. coastal) phytoplankton, microphytobenthos, *Zostera marina*, and riverine suspended particulate organic matter (RPOM). Primary consumers are estuarine-channel suspension feeders, estuarine-channel deposit feeders, estuarine-channel zooplankton, deep-bay deposit feeders, and deep-bay zooplankton. Values are given by medians and ranges (1–99 percentile).

Consumer groups	RPOM	*Phragmites australis*	Estuarine phytoplankton	Deep-bay phytoplankton	Microphyto-enthos	*Zostera marina*
**Estuarine channel**						
Suspesion feeder	1 / 0–5	16 / 10–20	81 / 74–86	1 / 0–7	0 / 0–3	0 / 0–1
Deposit feeder	3 / 0–16	10 / 0–30	68 / 27–100	9 / 0–54	4 / 0–26	2 / 0–12
Zooplankton	7 / 0–29	18 / 0–46	46 / 2–91	14 / 0–72	5 / 0–33	3 / 0–19
**Deep bay**						
Deposit feeder	2 / 0–17	6 / 0–38	3 / 0–22	69 / 22–97	14 / 0–49	3 / 0–16
Zooplankton	8 / 0–38	13 / 0–44	6 / 0–36	47 / 4–87	11 / 0–54	7 / 0–34

Supplementary Table 8 | Feasible contributions (%) of primary consumers to the diets of dominant motile crustaceans and fish in the estuarine channel. The IsoSource mixing model calculation was based on estuarine-channel zooplankton, estuarine-channel deposit feeders, deep-bay zooplankton, and deep-bay deposit feeders as end-members of pelagic and benthic baseline values in the estuarine channel and the deep bay, respectively. Values are given by medians and ranges (1–99 percentile).

	**Estuarine channel**		**Deep bay**	
Taxa	Zooplankton	Deposit feeders	Zooplankton	Deposit feeders
Crustaceans				
Charybdis japonica	8 / 0–32	15 / 0–50	15 / 0–54	58 / 29–81
Crangon hakodatei	7 / 0–32	4 / 0–19	49 / 8–89	38 / 11–60
Trachysalambria curvirostris	9 / 0–33	8 / 0–29	30 / 0–85	51 / 15–80
Fish				
Pleuronectes yokohamae	1 / 0–4	18 / 11–23	1 / 0–7	79 / 74–89
Leiognathus nuchalis	3 / 0–12	62 / 50–78	4 / 0–19	29 / 15–45
Konosirus punctatus	4 / 0–16	4 / 0–16	12 / 0–42	78 / 58–97

Supplementary Table 9 | Identification of trophic group of co-occurring dominant taxa. Trophic position of each taxon appeared in the estuarine channel and the deep bay is summarized in Table 1 and Fig. 7.

Trophic group	Estuarine channel	Deep bay
Primary consumer		
Suspension feeder		
Bivalve	*Corbicula japonica*	
Deposit feeder		
Benthic faunae	*Neanthes japonica*	*Melita* sp.
	Prionospio japonicus	*Xenophthalmus pinnotheroides*
	Capitella capitata	*Capitella capitate*
		Magelona japonica
		Sternaspis scutata
		Paraprionospio pinnata
		Chaetozone sp.
		Praxillella affinis
Predator		
Benthic faunae		*Glycera chirori*
		Sigambra tentaculata
		Scoletoma longifolia
Crustaceans	*Charybdis japonica*	*Oratosquilla oratoria*
	Crangon hakodatei	*Charybdis bimaculata*
	Trachysalambria curvirostris	*Parapenaeopsis tenella*
		Metapenaeus joyneri
		Trachysalambria curvirostris
		Portunus trituberculatus
		Palaemon gravieri
		Alpheus japonicus
		Alpheus digitalis
Demersal cephalopods		*Loligo japonica*
		Octopus variabilis
		Euprymna morsei
Pelagic fish	*Leiognathus nuchalis*	*Leiognathus nuchalis*
	Konosirus punctatus	*Thryssa kammalensis*
Demersal fish	*Pleuronectes yokohamae*	*Amblychaeturichthys hexanema*
		Cynoglossus joyneri
		Ctenotrypauchen microcephalus
		Johnius grypotus
Piscivorous fish		*Pennahia argentata*
		Okamejei kenojei

Supplementary Figures

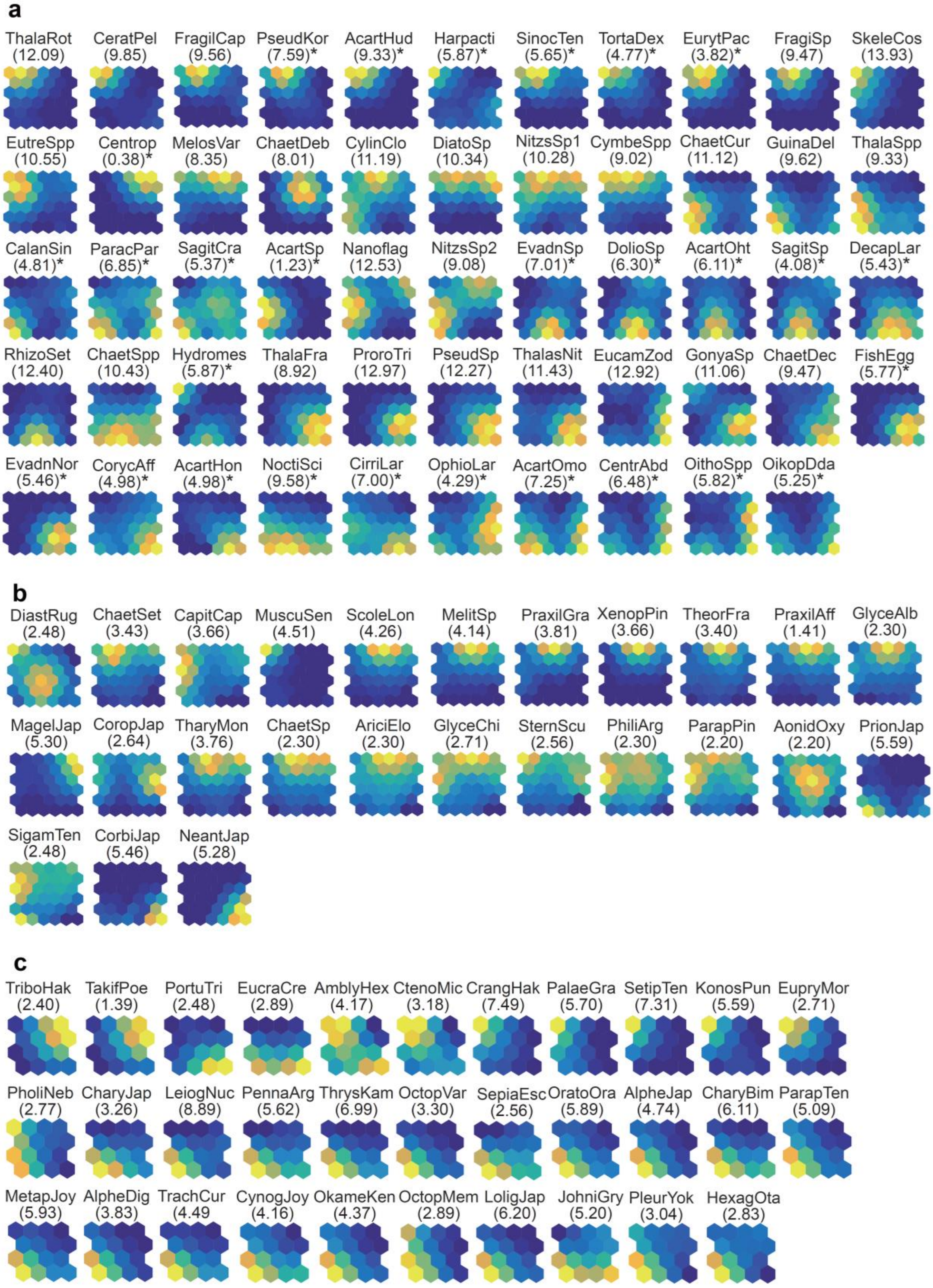

Supplementary Fig. 1 | Visualization of spatial distribution patterns of the SOM units.

Abundance and frequency data of common taxa are shown in Supplementary Table 1. The species name is abbreviated from the taxon list of Supplementary Table 1. The probability of occurrence of each taxon is visualized by the degree of colour (yellow = high probability of occurrence, blue = low probability of occurrence). Maximum values of log-transformed [ln(abundance + 1)] abundances of individual taxa are indicated in parentheses.

(**a**) Twenty-six phytoplankton and 28 zooplankton (*) taxa. As indicated by similar yellow and blue regions on the SOM map, some phyto- and zooplankton taxa exhibited similar distribution patterns with their maximum abundances in the upper left corner, revealing their close correlations. In contrast, phytoplankton taxa and nanoflagellates that occupied the upper right corner with their maximum values even showed high abundances in the upper left or lower left corner clusters. Some other phyto- and zooplankton taxa displayed typical distributions with their maximum abundances in the lower left, middle, and right corners, respectively.

(**b**) Twenty-five macrobenthic invertebrate taxa. As shown by SOM heatmaps generated from the 25 macrobenthic taxa in the training dataset, only two species (*Corbicula japonica* and *Neanthes japonica*) had similar heatmaps that showed their maximum abundances in the lower right corner of the SOM map, and only one species (*Prionospio japonicus*) exhibited the maximum in the lower left corner. In contrast, some macrobenthic taxa displayed maximum values in the upper middle and right corners and minimum values in the lower region.

(**c**) Seventeen nektonic invertebrates and 15 fish taxa. As manifested by the visual outputs of species from SOM training for the 32 nektonic taxa, only two fish species (*Tribolodon hakonensis* and *Takifugu poecilonotus*) showed typical distribution patterns in the upper right corner of the heatmaps, while two crabs (*Portunus trituberculatus* and *Eucrate crenata*) occurred with maximum values in the lower right corner. While some taxa (including shrimps, squids, and fish) set the maximum values in the upper left corner, many others displayed the maximum values in the lower left corner.
